# *Drosophila* Tempura, a Novel Protein Prenyltransferase α Subunit, Regulates Notch Signaling Via Rab1 and Rab11

**DOI:** 10.1371/journal.pbio.1001777

**Published:** 2014-01-28

**Authors:** Wu-Lin Charng, Shinya Yamamoto, Manish Jaiswal, Vafa Bayat, Bo Xiong, Ke Zhang, Hector Sandoval, Gabriela David, Stephen Gibbs, Hsiang-Chih Lu, Kuchuan Chen, Nikos Giagtzoglou, Hugo J. Bellen

**Affiliations:** 1 Program in Developmental Biology, Baylor College of Medicine, Houston, Texas, United States of America; 2 Department of Molecular and Human Genetics, Baylor College of Medicine, Houston, Texas, United States of America; 3 Jan and Dan Duncan Neurological Research Institute, Texas Children′s Hospital, Houston, Texas, United States of America; 4 Howard Hughes Medical Institute, Baylor College of Medicine, Houston, Texas, United States of America; 5 Medical Scientist Training Program, Baylor College of Medicine, Houston, Texas, United States of America; 6 Program in Structural and Computational Biology and Molecular Biophysics, Baylor College of Medicine, Houston, Texas, United States of America; 7 Department of Neurology, Baylor College of Medicine, Houston, Texas, United States of America; 8 Department of Neuroscience, Baylor College of Medicine, Houston, Texas, United States of America; Stanford University, United States of America

## Abstract

Vesicular trafficking plays a key role in tuning the activity of Notch signaling. Here, we describe a novel and conserved Rab geranylgeranyltransferase (RabGGT)-α–like subunit that is required for Notch signaling-mediated lateral inhibition and cell fate determination of external sensory organs. This protein is encoded by *tempura*, and its loss affects the secretion of Scabrous and Delta, two proteins required for proper Notch signaling. We show that Tempura forms a heretofore uncharacterized RabGGT complex that geranylgeranylates Rab1 and Rab11. This geranylgeranylation is required for their proper subcellular localization. A partial dysfunction of Rab1 affects Scabrous and Delta in the secretory pathway. In addition, a partial loss Rab11 affects trafficking of Delta. In summary, Tempura functions as a new geranylgeranyltransferase that regulates the subcellular localization of Rab1 and Rab11, which in turn regulate trafficking of Scabrous and Delta, thereby affecting Notch signaling.

## Abstract 2

A forward genetic screen in *Drosophila* looking for Notch signaling regulators identifies Tempura, a new and non-redundant protein prenyltransferase of Rab proteins.

## Author Summary

Notch signaling is an evolutionarily conserved signaling pathway that regulates many developmental processes. Abnormal Notch signaling activity can lead to numerous diseases and developmental defects. To better understand the regulation of this pathway, we performed a forward genetic screen for Notch signaling components that have not been previously identified in *Drosophila*. Here, we report the identification of an evolutionarily conserved protein, Tempura, which is required for Notch signaling-mediated lateral inhibition and cell fate determination of external sensory organs. We show that loss of *tempura* leads to mistrafficking of Delta and Scabrous, two important Notch signaling components. In addition, Rab1 and Rab11, two major coordinators of vesicular trafficking, are mislocalizaed in *tempura* mutants. We further show that Tempura functions as a subunit of a previously uncharacterized lipid modification complex to geranylgeranylate (a type of prenylation) Rab1 and Rab11. This post-translational modification is shown to be required for the proper subcellular localization and function of these Rabs. We find that dysfunction of Rab1 causes an accumulation of Delta and Scabrous in the secretory pathway and dysfunction of Rab11 further interferes with the trafficking of Delta. In addition to the known Rab geranylgeranyltransferse, our data indicate the presence of another functionally nonredundant Rab geranylgeranyltransferse, Tempura.

## Introduction

Notch signaling is an evolutionarily conserved pathway that plays a pivotal role in many developmental processes, including lateral inhibition, binary cell fate determination, and boundary formation [1],[2]. Aberrant Notch signaling is implicated in diseases such as Alagille syndrome, spondylocostal dysostosis (SCD), cerebral autosomal dominant arteriopathy with subcortical infarcts and leukoencephalopathy (CADASIL), and numerous types of cancer [3],[4]. Notch signaling depends on the direct contact between cells: the membrane-bound ligand, Delta (Dl) or Serrate, activates Notch on neighboring cells, resulting in proteolytic cleavages of Notch to generate a Notch intracellular domain (NICD) that activates the transcription of target genes [5].

**Figure 1:**
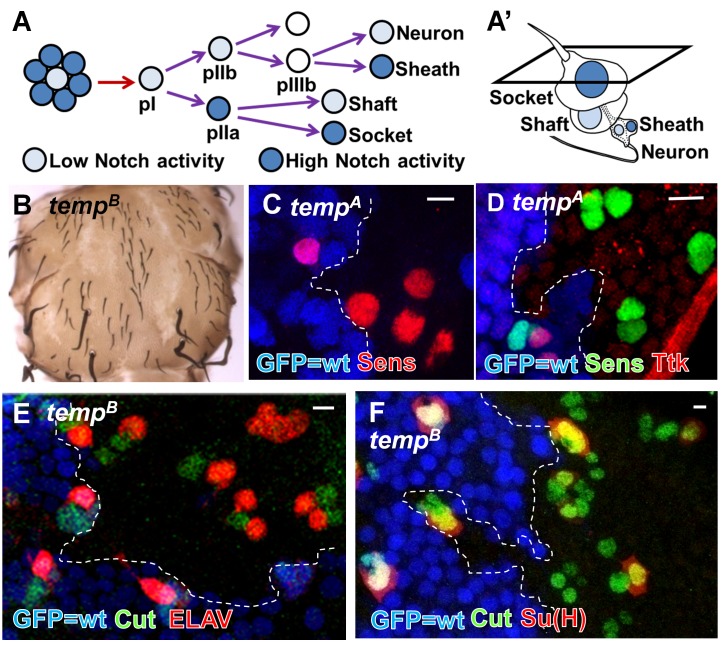
*temp* is essential for proper Notch signaling activity during ESO development. (A–A′) An ESO of *Drosophila* consists of four cells: shaft, socket, sheath, and neuron. During lateral inhibition, Notch ensures that only one SOP is selected from a proneural cluster. Subsequently, asymmetric Notch signaling activity also determines cell fates of the four descendants of the SOP. (B) *temp* mutant clones exhibit balding on an adult notum, a typical loss-of-Notch signaling phenotype. (C) At 12 h APF, the density of SOP (marked by Sens) is higher in *temp* mutant clones (GFP-negative), indicating a lateral inhibition defect. (D) At 19 h APF, Ttk, a Notch signaling effector strongly up-regulated in the pIIa cell, is lost in *temp* mutant sensory organs, indicating a loss of Notch signaling activity at this stage. (E) At 27 h APF, the presence of multiple neurons (marked by ELAV staining) per sensory cluster (marked by Cut staining) indicates that cell fate specification is impaired. (F) Su(H)-positive socket cells are lost in many mutant sensory organs, further indicating defects in cell fate decisions. Scale bars, 5 µm.

The developing adult external sensory organs (ESOs) on the *Drosophila* notum serves as a model system to study lateral inhibition and cell fate determination [6] and have led to the isolation of some Notch signaling components in forward genetic screens [7]–[10]. An ESO consists of four cells—shaft, socket, sheath, and neuron—which are derived from a single mother cell, the sensory organ precursor (SOP or pI) (Figure 1A and 1A′). Lateral inhibition ensures that only one SOP is selected from a proneural cluster. SOPs undergo several rounds of asymmetric cell division to generate four different cells and Notch signaling activity determines cell fates in each division (Figure 1A and 1A′). Loss of Notch signaling during lateral inhibition results in a higher density of ESOs, whereas its loss during cell fate determination causes ESO cells to take on neuronal fates, resulting in adult notum balding [11].

Notch signaling activity can be altered by defects in vesicular trafficking [12], coordinated by specific Rabs and their effectors. Rabs are small GTPases belonging to the Ras superfamily of small G proteins, which can switch between GDP-bound inactive and GTP-bound active forms [13]–[15]. Newly synthesized Rabs are prenylated with geranylgeranyl groups on C-terminal cysteines by a protein prenyltransferase (PPT) complex, a process required for their proper membrane localization and hence function [16]–[20]. PPTs are composed of αβ heterodimers and they add prenyl lipids (15-carbon farnesyl or 20-carbon geranylgeranyl groups) to cysteine residues located close to the C-termini of their substrates. Three PPTs have been identified so far: farnesyltransferase (FT), geranylgeranyltransferase I (GGTI), and the Rab geranylgeranyl-transferase [RabGGT, also known as geranylgeranyltransferase II (GGTII)] (Figure S1) [21],[22].

Here, we describe the isolation of mutations in a novel PPT α subunit repeat (PPTA) motif containing protein from an unbiased forward mosaic genetic screen for essential genes that affect Notch signaling. Due to its role in adding lipids to its substrates, we name this gene “*tempura*” (*temp*), based on a Japanese deep-fried dish. Our data show that Temp forms a new PPT to modify a small subset of Rabs, including Rab1, which has not previously been implicated in Notch signaling, and Rab11. Loss of *temp* leads to aberrant subcellular distribution of Rab1 and Rab11, which in turn leads to Notch signaling defects. In summary, we describe the function of a previously unidentified PPT, provide the first link between Rab1 and Notch signaling, and show that some Rabs are modified by two nonredundant PPTs. These data imply a complex regulation of Rabs by different PPTs that was not previously appreciated.

## Results

### *temp* Is Required for Notch Signaling Activity During ESO Development

To identify novel modulators of Notch signaling, we performed a forward genetic screen on the *Drosophila* X chromosome using ethyl methanesulfonate (EMS) [23]. We induced homozygous mutant clones of essential genes in the notum of otherwise heterozygous mutant animals with the FLP/FRT system [24], and screened for adult notum balding. We identified a novel complementation group named *temp*, consisting of seven alleles that exhibit a strong balding phenotype (Figure 1B).

To characterize the ESO development defects in *temp* mutant clones, we examined lateral inhibition and cell fate determination at different pupal stages. At 12 h after puparium formation (APF), the density of SOPs [marked by Senseless (Sens)] [25] is higher in *temp* mutant clones than in neighboring wild-type (wt) tissue, indicating a lateral inhibition defect (Figure 1C). To determine if cell fate specification is impaired, we assessed the expression of Tramtrack (Ttk), a downstream effector of Notch that is up-regulated in the signal-receiving pIIa cell but not in the signal-sending pIIb cell at 19 h AFP [26],[27]. We observed a loss of Ttk in *temp* mutant pIIa, indicating a loss of Notch signaling during cell fate determination at the two-cell stage (Figure 1D). At 27 h APF, when the four cells comprising an ESO are specified, many *temp* mutant ESOs contain multiple neurons (marked by the neuronal marker Embryonic Lethal Abnormal Vision, ELAV, [28] and lack socket cells, marked by Suppressor of Hairless, Su(H)) [29], indicating an alteration in cell fate (Figure 1A and 1E–F). This phenotype is not fully penetrant as 63% of the *temp^B^* mutant ESO cells are ELAV-positive, similar to what we observed previously for *dEHBP1* and *sec15* [8],[9], two players that affect vesicle trafficking and Notch signaling during ESO development. Although we observe some minor apoptosis in some *temp^A^* mutant clones (a strong allele; Figure S2A), there is no obvious apoptosis in *temp^B^* mutant clones (a less strong allele; Figure S2B). Overexpression of the antiapoptotic protein p35 in *temp^A^* mutant clones (Figure S2C) does not alter the phenotype (Figure S2D) when compared to *temp* mutant clones without p35 expression. In addition, there is no decrease in the number of sensory progenitor cells (marked by either Sens or Cut) (Figure 1C–F). These data indicate that lateral inhibition and cell fate transformation are due to loss-of-Notch signaling. Hence, *temp* is necessary for proper Notch signaling during the development of ESO lineage.

### *temp* Encodes a Protein with a PPTA Motif

**Figure 2:**
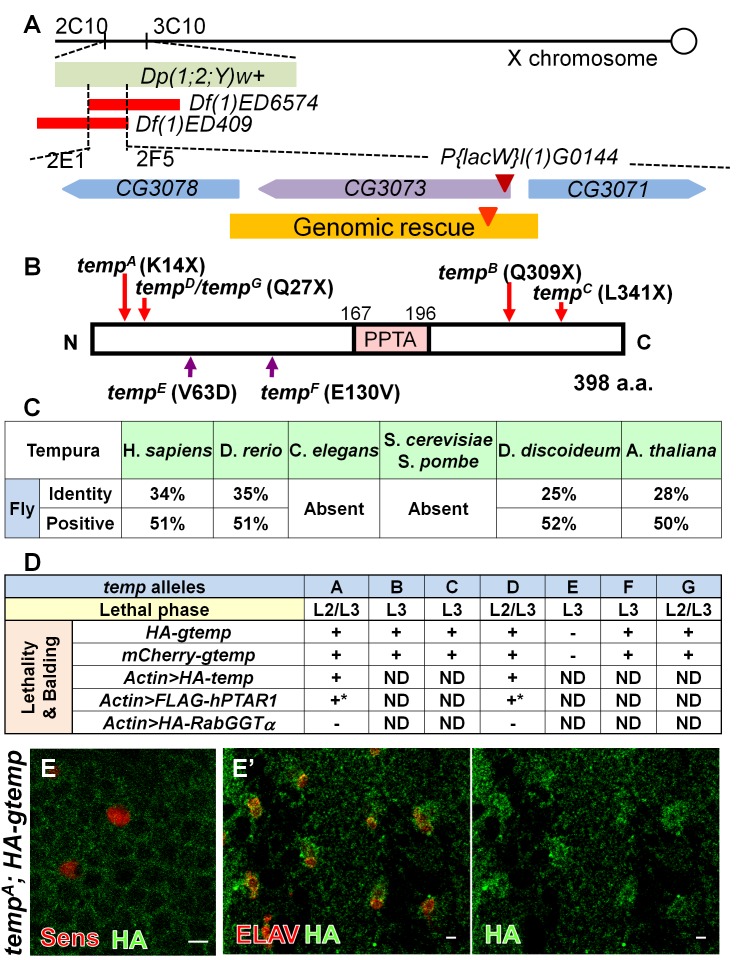
*temp* encodes a conserved PPTA motif-containing protein. (A) The lethality and phenotype of *temp* mutant is rescued by *Dp(1;2;Y)w+* (cytological regions 2C10–3C10) and fails to be complemented by *Df(1)ED6574* and *Df(1)ED409* narrowing the candidate region to 2E1 and 2F5. *P{lacW}l(1)G0144* [72], a *P-element* insertion (maroon triangle) in *CG3073*, fails to complement all *temp* alleles. The orange region indicates the extent of the genomic rescue constructs tagged with mCherry- or HA-tag (red triangle). (B) After sequencing the genomic region to which the *temp* alleles are mapped, we identified both nonsense mutations (*temp^A^*, *temp^B^*, *temp^C^*, *temp^D^*, and *temp^G^*) and missense mutations (*temp^E^* and *temp^F^*) within *CG3073*. This gene encodes a 398 a.a. protein containing a conserved PPTA subunit repeat motif (shown in pink). (C) *temp* homologs are conserved in many species queried. The percentage in the table is obtained via bl2seq. (D) Both lethality and balding phenotypes in all *temp* alleles (except for *temp^E^*) can be rescued by genomic and cDNA rescue constructs of *CG3073*. Because the lethality and balding of *temp^E^* can be rescued by the duplication, *Dp(1;2;Y)w^+^* (cytological regions 2C10–3C10) but not *gtemp*, this indicates that *temp^E^* carries a second lethal mutation in the 2C10–3C10 interval. Based on lethal phase analysis, the *temp^A^*, *temp^D^*, and *temp^G^* with early nonsense mutations are the most severe alleles. +, rescued; −, not rescued; ND, not determined. *Overexpression of human homolog of *temp*, *hPTAR1*, can rescue the balding phenotype in *temp* mutant clones on the notum, but its ubiquitous overexpression is toxic in flies even in the wt background. (E–E′) HA-tagged genomic *temp* (*HA-gtemp*) is expressed very weakly in the cytoplasm on the pupal notum during early ESO development (12 h APF) (E) and becomes slightly enriched in the sensory organs at the later stage (27 h APF) (E′). Scale bars, 5 µm.

Through duplication mapping [30], deficiency mapping [31], and complementation tests with existing lethal *P element* insertion lines [32], we mapped *temp* to *CG3073* (Figure 2A). This gene encodes a 398 amino acid (a.a.) protein containing a single small 29 a.a. PPTA motif which has only been found to be present in the α subunit of PPTs (Figure 2B). *temp* is evolutionarily conserved in most but not all species queried (Figure 2C), implicating that the function of the *temp* homolog might be assumed by another protein in species lacking this gene. The vertebrate homolog of *temp* is named PPTA containing protein 1 (*PTAR1*), but its biochemical or *in vivo* function has not yet been characterized.

The lethality and loss-of-Notch signaling phenotypes of *temp* are rescued by both genomic and cDNA rescue constructs (Figure 2A and 2D). Moreover, the human *PTAR1* cDNA can also rescue the ESO developmental defects in *temp* mutant clones (Figure 2D). These data indicate that the lethality and balding phenotypes in *temp* mutants are caused by mutations in *CG3073* and that the molecular function of *temp* is evolutionarily conserved between fly and human. To investigate the endogenous expression pattern of Temp, we attempted to generate several antibodies against Temp, but these were unsuccessful. We therefore examined the expression pattern of Hemagglutinin (HA)-tagged genomic constructs (*HA*–*gtemp*) in a *temp^A^* homozygous mutant background. We find that Temp is expressed weakly during early ESO development (Figure 2E) and that it is somewhat enriched in the cytoplasm of ESOs at the four-cell stage (Figure 2E′). *HA*–*gtemp* is expressed weakly and dispersed throughout the cytoplasm. Similarly, when we overexpress *HA*–*temp* cDNA using dpp–Gal4 in the wing disc, HA–Temp is also diffuse in the cytoplasm (Figure S3).

### *temp* Is Required for Scabrous Secretion

**Figure 3:**
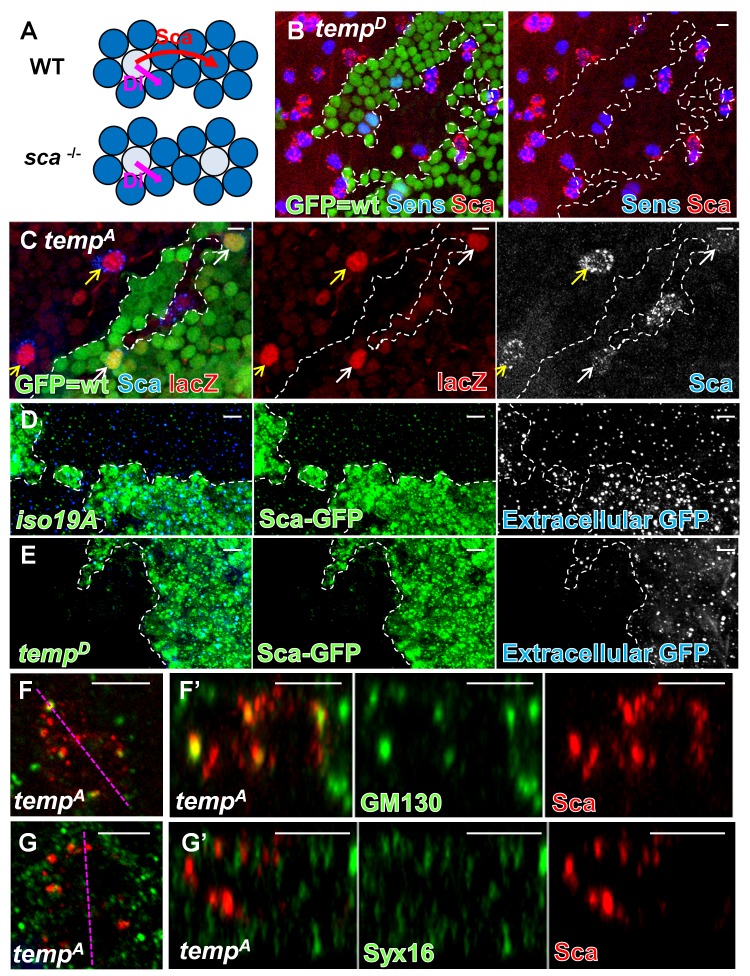
*temp* is required for Sca secretion in ESO in early secretory pathway. (A) Sca is secreted by SOPs to facilitate Notch signaling in nearby cells, preventing them from adopting SOP fates. *sca* mutants exhibit mild lateral inhibition defects similar to that of *temp* mutants. (B) The expression level of Sca is elevated in *temp* mutant ESOs (marked by Sens). (C) The expression level of *sca*–*lacZ*, as a readout for the transcription level of Sca, is similar between mutant (yellow arrows) and wt (white arrows) SOPs, indicating that an elevated level of Sca in *temp* mutant clones results from posttranscriptional up-regulation (the mutant SOPs in the middle small clone are out-of-plane). (D–E) Sca secretion assay: Sca–GFP fusion protein is expressed in either control or *temp* mutant clones (marked by strong GFP staining) using the MARCM strategy. The extracellular dots shown in the images are the extracellular staining for Sca–GFP, as they are secreted by the expressing cells and diffuses into the neighboring nonexpressing region. (D) Sca–GFP expressed in the wt (*y w FRT19A^iso^*) clone can be secreted to the neighboring region. (E) Sca–GFP expressed in *temp* mutant clones is absent in the neighboring wt region, indicating a secretion defect in *temp* mutant clones. (F–G′) Colocalization of Sca and Golgi markers. Each image is a projection of three optical slices. (F) Intracellular Sca puncta largely colocalize with GM130, a marker predominantly localized to the *cis*-Golgi, in *temp* mutant ESO. (F′) *z* axis view of (F). (G) Sca puncta do not colocalize with Syx16, a *trans*-Golgi marker, in *temp* mutant ESO. (G′) *z* axis view of (G). Scale bars, 5 µm.

The elevated SOP density in *temp* mutant clones (Figure 1C) is similar to what has been observed in *scabrous* (*sca*) mutants (Figure 3A) [33]. Sca is secreted by the SOP to facilitate Notch signaling in nearby cells to promote lateral inhibition [33]–[36]. It is rapidly secreted and degraded upon synthesis [37] and therefore is very difficult to detect in wt tissue on the notum (Figure 3B; GFP-positive cells). Interestingly, Sca is strongly up-regulated in *temp* mutant ESOs (Figure 3B; GFP-negative cells). A similar elevation of Sca in sensory organs is also observed in developing wing and eye imaginal discs (Figure S4A–B′), indicating that this elevation is not limited to the notum. Given that the lateral inhibition defect in *temp* mutant clones is similar to that of loss-of-function of *sca*, we hypothesized that Sca is produced, but that it fails to be secreted, and hence accumulates in *temp* mutant ESOs. To test this idea, we first determined whether the up-regulation of Sca in *temp* mutant is transcriptional or posttranscriptional. The expression of the *sca*–*lacZ* reporter [36],[38], a readout for *sca* transcription, is similar between *temp* mutant and wt ESO (Figures 3C and S4C–D), indicating that the level of Sca in *temp* mutant ESOs is posttranscriptionally up-regulated. To determine if Sca secretion is impaired, we developed a secretion assay by overexpressing a Sca–GFP fusion protein [39] in wt and *temp* mutant clones using the mosaic analysis with a repressible cell marker (MARCM) [40]. Sca–GFP can be secreted from wt clones into the neighboring area that does not produce Sca–GFP (Figure 3D). However, Sca–GFP produced in *temp* mutant clones fails to be secreted into the neighboring area (Figure 3E), indicating defective Sca secretion.

To determine where Sca accumulates intracellularly, we performed coimmunostaining of Sca and an array of subcellular organelle markers (Table S1). The Sca-positive puncta mainly colocalize with GM130 [41], a *cis*-Golgi marker, but not with Syntaxin 16 (Syx16) [42], a *trans*-Golgi marker (Figure 3F–G′). Therefore, in *temp* mutant cells, Sca accumulates in a GM130-positive compartment and cannot be secreted, which in turn contributes to the defect in Notch-signaling–mediated lateral inhibition.

### *temp* Is Required for Proper Localization of dEHBP1 and Dl

**Figure 4:**
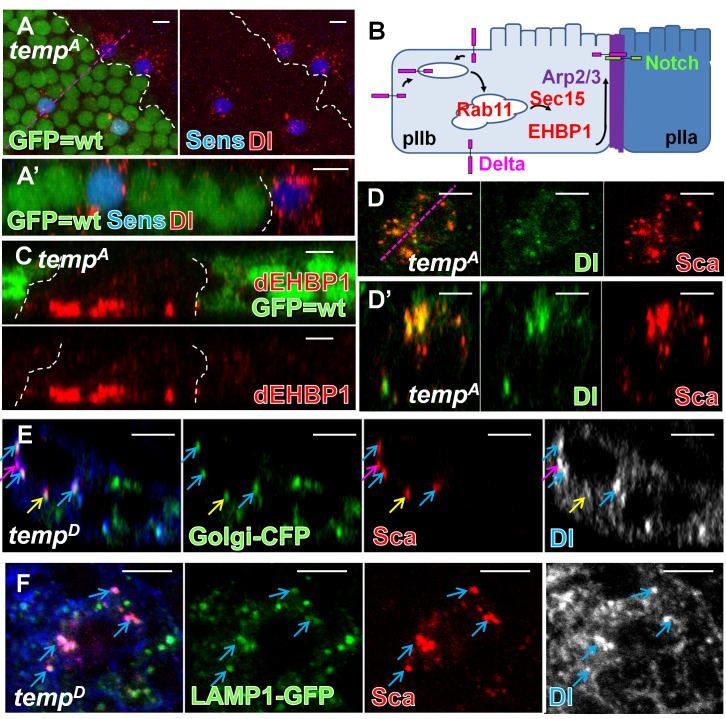
*temp* is required for proper localization of dEHBP1 and Dl during ESO development. (A) At 16 h APF, more Dl puncta are present in the *temp* mutant SOPs (marked by Sens) compared to the wt ones. (A′) *z* axis view of (A). (B) Dl recycling model at two-cell stage: Recycling of Dl in the pIIb is required for proper Notch signaling activity in the pIIa. After endocytosis, Dl is recycled through Rab11–Sec15–dEHBP1-dependent recycling route along the ARS to the apical interface between pIIb and pIIa, where Dl is thought to activate Notch receptor on the pIIa. (C) dEHBP1 accumulates basally in *temp* mutant clones. (D–D′) Many Dl puncta colocalize with Sca in *temp* mutant ESO. (D) Single *x*–*y* section. (D′) Single *z* section. (E) Dl and Sca puncta largely colocalize with Golgi–CFP in *temp* mutant clones with some distribution dynamics (single *z* section). Blue arrow, colocalized proteins locate to the Golgi–CFP-positive compartments; pink arrow, colocalized proteins not associated with the Golgi complex; yellow arrow, Sca, but not Dl, colocalizes with Golgi–CFP-positive compartments. (F) Some Dl and Sca puncta colocalize with LAMP-1–GFP in *temp* mutant clones (single *z* section). Scale bars, 5 µm.

Since Sca is primarily involved in the lateral inhibition process, other proteins are likely to contribute to the cell fate specification defect in *temp* mutant clones (Figure 1E and 1F). Cell fate determinants, including the adaptor protein Numb [26],[43] and the E3 ligase Neuralized [44]–[46], are asymmetrically segregated during the division of the pI cell to bias Notch signaling between the pIIa and pIIb. We did not observe obvious defects in the localization of either protein, suggesting that asymmetric segregation of cell fate determinants is not affected (not shown). Although the expression and localization of Notch is not affected (Figure S5A–C), the number of Dl-positive puncta is increased in *temp* mutant sensory organs (Figure 4A and 4A′). We previously proposed that recycled Dl travels to the apical actin-rich structure (ARS) localized between the pIIa and pIIb at the two-cell stage (Figure 4B) [10]. This process is necessary for proper Notch signaling activation and requires the Arp2/3 complex as well as the vesicle trafficking regulators Sec15 and dEHBP1, two binding partners of Rab11 [8],[9],[47],[48]. Mutations in these genes have been shown to exhibit cell fate defects and notum balding, similar to the loss of *temp*. While the ARS is properly formed (not shown) and apical-basal polarity is not affected in *temp* mutant clones (Figure S5E), we found that dEHBP1 accumulates basally (Figure 4C), similar to what is observed in *sec15* mutants [8]. In *dEHBP1* mutant ESOs, the level of Dl is reduced at the cell surface [8], a feature that we also observe in about half (yellow arrows) of the ESOs in *temp* mutant clones (Figure S5D). These data indicate that Dl accumulates intracellularly and that the mislocalization of EHBP1 may at least partially contribute to the Dl trafficking defect in *temp* mutant ESOs. Additionally, we found that many of the Dl and Sca puncta colocalize in *temp* mutant ESO, indicating that some of them are trapped in the same intracellular compartments (Figure 4D and 4D′). The majority of colocalized proteins are in the Golgi complex (Figure 4E, blue arrows). However, some of the Dl- and Sca-positive puncta colocalize with a late endosomal and lysosomal marker LAMP1–GFP (Figure 4F), suggesting that defects in the secretory pathway may cause some Sca and Dl to be sorted to the endo-lysosomal pathway for degradation. Therefore, mistrafficking of Dl is also likely to contribute to both lateral inhibition and cell fate defects in *temp* mutants.

### Temp Is a New α Subunit of RabGGT Complex

**Figure 5:**
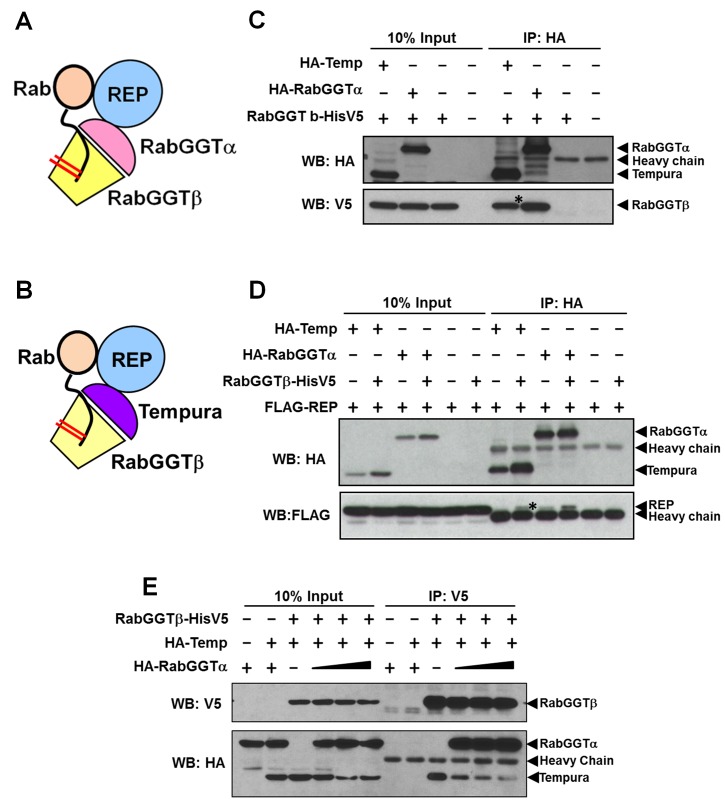
Temp is a novel α subunit of RabGGT complex. (A) The canonical RabGGT complex is composed of α and β subunits. After REP recruits Rabs, RabGGT adds geranylgeranyl groups to cysteine residues located close to the C-terminal of Rabs. (B) Proposed model of Temp function: Temp functions as an alternative α subunit in RabGGT complex to prenylate specific Rabs with geranylgeranyl groups. (C–D) CoIP experiments in *Drosophila* S2 cells reveal interactions between Temp, RabGGTβ, and REP. *Drosophila* RabGGTα (PTAR3) also interacts with RabGGTβ and REP, shown as a positive control. Note that the expression level of Temp is increased in the presence of RabGGTβ, suggesting that Temp is stabilized through binding to RabGGTβ. (C) Temp interacts with RabGGTβ. (D) Temp interacts with REP. (E) Temp and RabGGTα compete for RabGGTβ: The amount of RabGGTβ is kept at a low level to serve as a limiting factor in complex formation. The transfection levels of both RabGGTβ and Temp are kept the same, whereas the amount of RabGGTα is increased gradually. The same amount of RabGGTβ is pulled down by α-V5 beads. The interaction between RabGGTβ and Temp (pulled down by RabGGTβ) is reduced when the amount of RabGGTα is increased. To show all the bands using the same exposure time, the signal for RabGGTα is saturated. Note that the expression level of Temp is also reduced when it lost its binding to RabGGTβ.

Protein prenylation regulates protein targeting and activity of numerous proteins [21],[22]. Given that Temp contains a PPTA motif and that *temp* mutants affect protein trafficking, Temp may regulate the prenylation of proteins involved in vesicular trafficking. Indeed, RabGGTβ was identified as a potential interactor for *Drosophila* Temp in a high-throughput yeast-2 hybrid screen [49]. RabGGTβ forms a complex with RabGGTα [also called PPTA containing protein 3 (PTAR3)] and Rab escort protein (REP) to geranylgeranylate Rabs (Figure 5A) [16],[50]–[54], which are major coordinators of vesicle trafficking [13],[14]. We therefore tested if Temp may act as an alternative α subunit of the RabGGT complex (Figure 5B). Indeed, we found that Temp can interact with RabGGTβ and REP in coimmunoprecipitation (coIP) experiments in *Drosophila* S2 cells (Figure 5C and 5D). In the presence of RabGGTβ, the expression level of Temp is increased, suggesting that Temp is stabilized by RabGGTβ. To assess whether Temp is an additional subunit of the original RabGGT complex or an alternative α subunit in a new RabGGT complex, we performed a RabGGTβ competition assay (Figure 5E). We pulled down the same amount of RabGGTβ and found that its binding to Temp is reduced when the amount of RabGGTα increases, indicating that Temp and RabGGTα compete for RabGGTβ. Note that the total level of Temp is also decreased when it cannot bind to RabGGTβ. These data suggest that RabGGTβ can form two RabGGT complexes: a canonical complex with RabGGTα and a novel complex with Temp.

### Impairing Rab1 and Rab11 Functions Phenocopies Loss of *temp*

Rabs are the only known substrates of the canonical RabGGT complex (RabGGTα–RabGGTβ) [16],[21],[55]. Hence, the targets of the Temp–RabGGTβ complex may also be Rabs. To identify the substrate Rabs responsible for the loss-of-Notch signaling phenotypes in *temp* mutants, we performed a genetic screen and overexpressed a subset of UAS–YFP dominant-negative Rab (*DN-Rab*) lines [56] in wing imaginal discs and pupal notum to screen for Sca accumulation and balding phenotypes similar to *temp* mutant clones, respectively (Table S2). Because Sca secretion defects in *temp* mutants are not restricted to the notum, we tested Rabs that are broadly expressed [57] and those involved in protein secretion [14].

**Figure 6:**
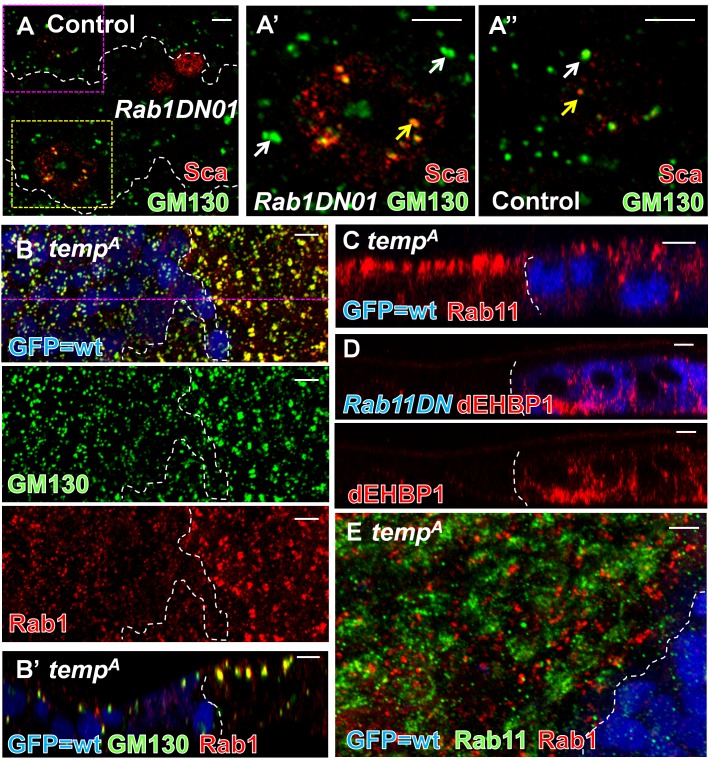
Dysfunction of Rab1 and Rab11 each phenocopies some features of loss of function of *temp*. (A) Sca colocalizes with expanded GM130-positive compartment in *Rab1DN*-expressing cells (single section). (A′) Enlargement of the yellow boxed *Rab1DN*-expressing region in (A). (A″) Enlargement of the pink boxed control region in (A). (B–B′) Rab1 and its effector GM130 exhibit an altered distribution in *temp* mutant clones. (B) Projection. (B′) Single *z* section of (B). (C) Rab11 mislocalizes apically in *temp* mutant clones. (D) dEHBP1, a Rab11 interactor, accumulates basally in *DN-Rab11*-expressing cells, similar to what has been observed in *sec15* mutant and *temp* mutant clones. (E) Rab1 and Rab11 are present in distinct compartments and do not colocalize with each other in *temp* mutant clones. Scale bars, 5 µm.

Among the 15 Rabs tested for Sca accumulation, only overexpression of *DN-Rab1* reproduces key features of *temp* mutant cells. It causes a strong Sca accumulation in ESO in third instar wing discs (Table S2), consistent with what we observed in *rab1* homozygous mutant clones (Figure S6A) [58]. We also observe a co-accumulation of both Dl and Sca in *DN-Rab1*-expressing ESOs on the pupal notum (Figure S6B). Similar to *temp* mutants, large Sca puncta colocalize with enlarged GM130 compartments [59] when we overexpress *DN-Rab1* (Figure 6A–A″). Most importantly, Rab1 accumulates in enlarged GM130-positive compartments in *temp* mutant clones (Figure 6B and 6B′), suggesting that Temp regulates the subcellular distribution of Rab1. Together, these data suggest that Rab1 may be a substrate for Temp. Therefore, loss of *temp* leads to aberrant localization of Rab1 and its effector GM130, which in turn causes an accumulation of Sca and Dl.

Because dysfunction of Rab1 only leads to minor adult balding (Table S2 and Figure S6C), the cell fate transformations observed in *temp* mutant cells may be contributed by the dysfunction of other Rab(s). Among the 10 broadly expressed Rabs [57], we found that expression of *DN-Rab5* and *DN-Rab11* lead to strong balding (Table S2). Because we observed aberrant subcellular localization of Rab11 (Figure 6C), but not Rab5 (not shown), in *temp* mutant clones, we focused on Rab11, which functions in Dl recycling during ESO development [9],[47]. In *temp* mutant cells, Rab11 is enriched apically (Figure 6C). This apical clustering is likely due to mislocalization of Rab11 because the protein level of Rab11 is not changed in *temp* mutant animals (Figure S6D).. Cells that express *DN-Rab11* exhibit an accumulation of Dl but not of Sca (Figure S6E and S6F), indicating that Rab11 regulates trafficking of Dl but not of Sca. In addition, dEHBP1, a Rab11 binding partner [8], aberrantly accumulates basally in *DN-Rab11*-expressing notum (Figure 6D), similar to *temp* mutants (Figure 4C). Therefore, loss of *temp* causes an aberrant localization of Rab11, which leads to a mislocalization of dEHBP1 and mistrafficking of Dl. These data indicate that Rab11 may also be a substrate of Temp.

Because *temp* mutant clones exhibit an altered distribution of both Rab1 and Rab11, we tested whether they are misdistributed to the same intracellular compartment. Coimmunostaining of Rab1 and Rab11 reveals that they mostly do not overlap in *temp* mutant clones (Figure 6E), suggesting that additional factors regulate their aberrant subcellular distribution in the absence of *temp.*

### Rab1 and Rab11 Are Substrates of the Temp–RabGGTβ Prenyltransferase Complex

**Figure 7:**
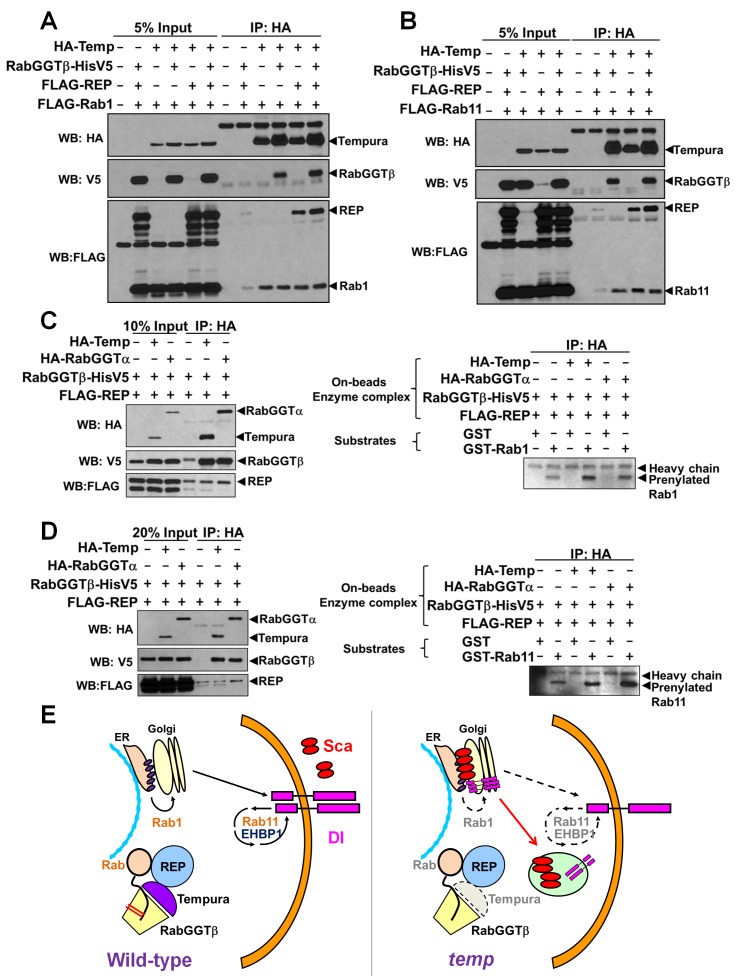
Rab1 and Rab11 are substrates of the Temp–RabGGTβ–REP complex. (A–B) Co-IP assay in S2 cells: Temp interacts with Rab1 (A) and Rab11 (B). The interaction can occur without the transfection of RabGGTβ and REP. This is possibly due to the endogenous expression of these two proteins in S2. The very weak pulled down band of Rab1/Rab11 in the absence of Temp is due to nonspecific binding to α-HA beads. (C–D) *In vitro* prenylation assays: (C, Left) Western blot result of input and IP for the prenylation assay. Because the whole pull-down process is performed in the prenylation buffer, which is not an optimal condition for IP, some nonspecific binding is present in the control. (C, Right) GST–Rab1 can be prenylated with geranylgeranyl groups in the presence of Temp and RabGGTα. The prenylation efficiency for Rab1 is much higher with Temp than with RabGGTα. The weak band in the control condition might be due to endogenous activity. (D, Left) Western blot result of input and IP for the prenylation assay. (D, Right) GST–Rab11 can be prenylated with geranylgeranyl groups in the presence of Temp and RabGGTα. The prenylation efficiency for Rab11 is higher with RabGGTα than with Temp under this substrate concentration. The weak band in the control condition might be due to endogenous activity. (E) Model of the role of Temp in Notch signaling: Temp functions in a new RabGGT complex. Rab1 and Rab11 are two substrates of this new RabGGT complex. The trafficking and localization of Notch signaling components are regulated by Rab1 and Rab11. Thus, in the absence of Temp, Rab1 and Rab11 are mislocalized, which in turn causes mistrafficking of Sca and Dl, resulting in the Notch signaling defects.

As shown previously, Temp can form a new RabGGT complex with RabGGTβ and interact with REP (Figure 5B–D). If Rab1 and Rab11 are substrates of this complex, they should physically interact with Temp. Indeed, Temp binds to Rab1 and Rab11 in coIP experiments in S2 cells (Figure 7A and 7B, last lanes). Temp and these Rabs can also be pulled down without cotransfecting RabGGTβ or REP, possibly because of the presence of these proteins in S2 cells. We knocked down the REP and RabGGTα proteins in S2 cells using RNAis that we designed. Unfortunately, these cells grow very slowly and most die, suggesting that *REP* and *RabGGTα* are required for the viability of S2 cells. These data are in agreement with our observation that *RabGGTα* mutant cells are lethal *in vivo* (unpublished data). To determine whether Temp can prenylate Rab1 and Rab11 with geranylgeranyl groups, we performed an *in vitro* prenylation assay [60]. We cotransfected REP, RabGGTβ, and HA–Temp or HA–RabGGTα into S2 cells and pulled down the enzyme complex with anti-HA beads (Figure 7C and 7D, left panels). We also purified GST–Rab1 and GST–Rab11 as unmodified substrates from bacteria. The prenylation assays were performed by adding GST–Rab1 or GST–Rab11 to biotin-labeled geranylgeranyl groups and the anti-HA beads loaded with the enzymatic complex (HA–Temp–RabGGTβ–REP or HA–RabGGTα–RabGGTβ–REP). In the absence of Temp and RabGGTα, we observe weakly prenylated bands, likely due to nonspecific pull-down of endogenous RabGGT complexes (Figure 7C and 7D). However, in the presence of Temp or RabGGTα (positive control) the prenylated band is obviously enhanced (Figure 7C and 7D, right panels). These data indicate that the complexes containing Temp can prenylate Rab1 and Rab11. Temp seems to be more efficient in prenylating Rab1, whereas RabGGTα seems to be more efficient in prenylating Rab11, indicating that they may have different substrate preferences. In conclusion, our data show that Temp can form a novel PPT complex to prenylate Rab1 and Rab11.

## Discussion

We isolated a novel Notch signaling player, *temp*, whose loss causes defects in lateral inhibition and cell fate determination of ESO from an unbiased genetic screen. *temp* encodes an unstudied protein with a 29 a.a. PPTA motif implicated in protein prenylation. Here, we show that Temp forms a complex with RabGGTβ and REP to prenylate Rab1 and Rab11. Loss of *temp* leads to an aberrant subcellular distribution of Rab1 and Rab11. Loss-of-function of Rab1 causes an accumulation of Sca and Dl, whereas loss-of-function of Rab11 further contributes to the Dl trafficking defect. Together, our data indicate that Temp functions as the α subunit of a new RabGGT complex that modulates Notch signaling via Rab1 and Rab11.

Although the role of Rab11 in Notch signaling pathway has been previously documented [47], our data indicate that Rab1 is required for proper trafficking of Sca and Dl. To our knowledge, this is the first time that Rab1 has been linked to Notch signaling. Sca trafficking is mainly affected by Rab1, whereas Dl trafficking is affected by both Rab1 and Rab11. Because null alleles of both Rabs are cell lethal (not shown), but *temp* mutant clones are not, the loss of Rab1 and Rab11 function can only be partial in the *temp* mutant clones. This suggests that the other α subunit, RabGGTα, still prenylates a portion of Rab1 and Rab11 that play a role in other cellular processes in the absence of *temp*, consistent with the prenylation assay results (Figure 7C and 7D). In addition, overexpression of constitutively active (CA) forms of Rab1 and Rab11 (Rab1CA and Rab11CA) in *temp* mutant clones does not alter Sca accumulation (Figure S7A and S7B) nor does it alter cell fate transformation (Figure S7C and S7D). This suggests that loss of *temp* is epistatic to the constitutively active forms of Rabs. These genetic interaction data are consistent with our model as constitutively active forms of Rab must be properly prenylated [18].

In *temp* mutant clones and notum cells expressing *DN-Rab1*, Sca mostly accumulates in GM130-positive compartments, traditionally considered as *cis*-Golgi. However, because Rab1 functions in ER-to-Golgi trafficking [13],[14], loss of Rab1 is expected to prevent cargo from entering the Golgi apparatus. This may be because the *cis*-Golgi is closely associated with the ER exit sites (tER) to form the “tER–Golgi unit” [61], which is optically difficult to distinguish from the cis-Golgi compartment. Indeed, a number of Golgi proteins including GM130, GRASP, and p115 localize at tER sites in S2 cells [61]–[63]. For example, GRASP also colocalizes with Sca in *temp* mutants (Figure S4E). Therefore, loss of *temp* or its target, Rab1, causes Sca to accumulate in GM130-positive compartments corresponding to the *cis*-Golgi, the tER, or an intermediate compartment between the two.

In yeast and cultured mammalian cells, Rabs that lack proper geranylgeranylation diffuse in the cytoplasm [17],[18]. Surprisingly, we find that the distribution of Rab1 and Rab11 in *temp* mutants is restricted to specific subcellular compartments: Rab1 colocalizes with enlarged GM130 compartments (Figure 6B) whereas the majority of Rab11 is apically enriched (Figure 6C). Given that Rab1 and Rab11 do not diffuse in the cytoplasm and that they do not redistribute to the same microdomains (Figure 6E), we propose that the subcellular localization of Rab1 and Rab11 is in part determined by different/other Rab binding partners upon reduction of proper prenylation in *temp* mutants.

The fly and human genomes both contain three genes that encode proteins containing the PPTA motif: PTAR1/Temp, PTAR2/FTα, and PTAR3/RabGGTα. PTAR2 forms PPTs with two different β subunits, whereas PTAR3 forms a RabGGT with RabGGTβ (Figure S1) [21],[22],[52]–[54],[64]. We show that PTAR1 and RabGGTβ form an alternative RabGGT complex to prenylate Rab1 and Rab11. This raises an interesting question: what is the labor distribution of RabGGTα–RabGGTβ and Temp–RabGGTβ complexes? Because the *PTAR1* (*temp*) homolog is absent in some species like *S. cerevisiae* and *C. elegans*, but is present in *Dictyostelium* and *Arabidopsis* (Figure 2C), it is likely that it was lost in some evolutionary branches and that its function is covered by *PTAR3* in these species. While we can obtain large homozygous mutant clones with *temp* null alleles, we find that homozygous *RabGGTα* mutant clones in the thorax and eyes are cell lethal (not shown). Hence, we speculate that Temp has evolved to play a more specific role to prenylate a subset of Rabs, whereas RabGGTα is able to modify most, if not all, Rabs [16],[21],[55] yet is not sufficient for proper trafficking of Scabrous and Delta. Indeed, although both Temp and RabGGTα can prenylate Rab1 and Rab11 *in vitro* with different efficiency (Figure 7C and 7D), expression of fly RabGGTα fails to rescue the ESO phenotypes in *temp* mutant clones (Figure 2D). Moreover, expression of fly *temp* does not alleviate the cell lethality in *RabGGTα* mutant clones (data not shown). These data indicate that the functions of the two RabGGT complexes are nonredundant *in vivo* and that the functions of the two RabGGT complexes towards different Rabs may also be regulated in a tissue-specific manner through unknown interaction partners and/or posttranslational modifications *in vivo*. This tissue-specific regulation is supported by the gene expression data from FlyAtlas (Figure S8) [65],[66]. *RabGGTα* mRNA is transcribed ubiquitously at moderate levels and the expression pattern of *temp* mRNA is much more restricted to the nervous system with high levels in thoracic-abdominal ganglion cells. This suggests that Temp plays a role in the nervous system, including ESO development.

As major coordinators of vesicular trafficking, Rabs are crucial for maintaining normal cellular function and misregulation of some Rabs results in cellular dysfunction. Indeed, dysfunction of some Rabs and their prenylation factors are implicated in several diseases [13],[55],[67]. For example, Choroideremia, an inherited retinal degenerative disease, is caused by mutations in REP1. On the other hand, Rab1 is hijacked by the pathogen *Legionella pneumophila* during infection to support the bacterium with the ER-to-Golgi secretory system [68]. In various cancers, the expression level of numerous Rabs, including Rab1 and Rab11, is up-regulated. Up-regulation of Rab11 family members (Rab11A/11B/25) is associated with more aggressive prostate, ovarian, and breast cancers [13],[55]. Finally, toxins produced by *Bacillus anthracis* inhibit Rab11 and Sec15, which in turn reduce Notch signaling activity in both flies and mammalian endothelial cells [69], suggesting a possible role for Temp in aberrant Notch signaling induced by bacterial infection.

## Materials and Methods

### Fly Strains, Transgenesis, and Crossing Schemes

The following stocks were used in this study: (1) isogenized *y w FRT19A (y w FRT19A^iso^* or *iso19A)*, (2) *Df(1)JA27/FM7c Kr-GFP*, (3) *w sn FRT19A; Ubx-FLP(2)*, (4) *cl(1) Ubi-GFP FRT19A/FM6; Ubx-FLP(2)*, (5) *Ubx-FLP tub-GAL80 FRT19A; Actin-GAL4 UAS-CD8::GFP* (MARCM line), (6) *hs-FLP Ubi-GFP FRT19A* (D. Bilder), (7) *y w; P{lacW}sca^A2–6^*(*sca-lacZ* reporter) [36],[38], (8) *y w; UAS-Sca-GFP/Cyo* (Sca-GFP used in secretion assay) [39], (9) *w; UAS-CFP-Golgi(2)* [70], (10) *w; UAS-Lamp1-GFP; M3–12/S-T* (C-K Yao) [71], (11) *y w; T(2;3)ap[Xa]/SM5; TM3, Sb*, *ER-YFP* [*19*], (12) *C96-Gal4/(TM3, Sb)*, (13) *Dp(1;2;Y)w+*, (14) *w Df(1)ED6574/FM7h*, (15) *w Df(1)ED409/FM7h*, (16) *P{lacW}l(1)G0144 w/FM7c* [72], (17) *FRT82B dar6[12-3-73]/TM3, Sb* [58] (a severe loss-of-function allele for Rab1), (18) *y w Ubx-FLP; FRT82B M* [*19*] *Ubi-GFP*, (19) *y w; P{w[+mW.hs] = FRT(w[hs])}2A P{ry[+t7.2] = neoFRT} 82B PBac{SAstopDsRed}LL03248 P{y[+t7.7] ry[+t7.2] = Car20y}96E/TM6B, Tb* [73], and (20) *UAS-p35* (a kind gift from Andreas Bergmann).

The forward genetic screen that identified alleles of *temp* was performed as previously described [23]. A total of 577 stocks have wing notching or notum balding phenotypes and were mapped through X chromosome duplication mapping, deficiency mapping, and complementation tests.

Genomic and cDNA rescue transgenic flies were generated by *phiC31*-mediated transgenesis at *vk33* or *vk37* docking sites [74]. Phenotypic rescue was assessed in *temp* mutant background either in whole animal or by overexpression in *temp* MARCM clones. *UAS*–*YFP*–*DN-Rab* flies [56] were crossed to *C96*–*Gal4* [75], which can drive ectopic expression of UAS transgenes around the wing margin, and used for immunostaining against Sca in the third instar larval wing disc. We overexpressed *UAS*–*GFPnls* as a negative control.

We used the MARCM strategy in the balding screen and to assess Sca secretion and mark subcellular compartments. Because these transgenes encode GFP/YFP/CFP fusion proteins, we crossed out *Actin*–*Gal4, UAS CD8::GFP* in MARCM line, which was then combined with the transgene of interest and subsequently crossed to either *y w FRT19A^iso^; Actin*–*Gal4/Cyo* or *y w temp FRT19A; Actin*–*Gal4/Cyo* (*temp^A^* and *temp^D^*) for performing experiments in control and mutant genetic backgrounds, respectively. Stocks were maintained at RT and crosses were performed at 25°C.

### Genomic and cDNA Constructs

A genomic rescue construct was constructed by PCR amplification of a 4.5 kb amplicon spanning *CG3073* locus and cloned into pattB [76]. We added N-terminal HA or mCherry tags to the genomic rescue construct via conventional cloning methods. cDNA of *RabGGTβ* was constructed in the pMT vector, while human *PTAR1(hPTAR1)*, *temp*, *RabGGTα*, *REP*, *rab1*, and *rab11* were cloned into pUASTattB [76] with N-terminal HA and FLAG tags. In addition, *rab1* and *rab11* were cloned to pGEX vectors (GE Healthcare). Cloning and DNA purification were performed based on standard protocols. Enzymes are from NEB, and DNA purification kits are from Invitrogen and Qiagen. All constructs were sequenced before injection or transfection.

### Dissection, Immunostaining, and Imaging

For notum immunostaining, fly pupae were aged until the indicated time points at 25°C. For the wing disc staining, we dissected third instar larvae. Dissection and immunostaining were performed as previously described [8]. Primary antibodies were used at the following dilutions: mouse α-Rab11 1∶100 (BD Biosciences), rabbit α-Rab11 1∶1,000 [69], mouse α-Rab1 1∶500 [77], guinea pig α-Boca 1∶1,000 [78], guinea pig α-Hrs 1∶600 [79], mouse α-Dl 1∶200 (DSHB) [80], guinea pig α-Dl 1∶1,000 [81], mouse α-NICD 1∶200 (DSHB) [82], mouse α-NECD 1∶100 (DSHB), chicken α-GFP 1∶1,000 (Abcam), rabbit α-GFP 1∶500 (Invitrogen), rabbit α-GM130 1∶500 (Abcam), rabbit α-Syx16 1∶500 (Abcam), rabbit α-Neur 1∶600 [83], rabbit α-beta-Galactosidase 1∶500 (Cappel), rabbit α-Numb 1∶1,000 [43], rabbit α-GRASP55 1∶500 [84], guinea pig α-Sens 1∶1,000 [25], rat α-ELAV 1∶500 (DSHB) [85], mouse α-Cut 1∶500 (DSHB) [86], rat α-Su(H) 1∶500 [29], rat α-*D*E-cadherin 1∶50 (DSHB) [87], and rat α-Ttk69 1∶500 [88]. Alexa 488–conjugated (Invitrogen) and Cy2-, Cy3-, Cy5-, or DyLight649-conjugated secondary antibodies (Jackson ImmunoResearch) were used at 1∶200. Samples were mounted in Slowfade reagent (Invitrogen). All confocal figures were acquired with confocal microscope (LSM510; Carl Zeiss) using Plan Apochromat 40× NA 1.4 and Plan Apochromat 63× NA 1.4 objectives (Carl Zeiss), followed by processing in LSM software, ImageJ, and Photoshop (Adobe).

For Sca secretion assays, we assess extracellular Sca–GFP by staining for rabbit α-GFP without any detergents. Then, we permeabilize the notum tissue with 0.1% Triton-PBS and perform a staining for the total GFP using chicken α-GFP antibody. To costain both extracellular and total Dl in *temp* mosaic notums, we first stain extracellular Dl using mouse α-Dl antibody without detergents. Then we permeabilize the noum with 0.1% Triton-PBS and stain for the total Dl with the guinea pig α-Dl antibody. For Notch extracellular staining, the notum tissue is first stained with mouse α-NECD antibody without any detergents. Then, we permeabilize the notum tissue with 0.1% Triton-PBS and perform the later steps of immunostaining similarly.

### Cell Culture, CoIP, and Western Blot

S2 cell line was cultured in Schneider's media (Gibco) with 10% fetal bovine serum (FBS) (Sigma-Aldrich) and antibiotics mix (penicillin and streptomycin, Invitrogen) at RT. The transfection was performed using Effectene (Qiagen) according to the manufacturer's instructions. Proteins were expressed using Actin–GAL4/UAS system or pMT inducible system (Invitrogen). In co-IP experiments, cells were harvested 48 h after transfection and lysed on ice in lysis buffer [50 mM Tris-HCl pH 7.5, 150 mM NaCl, 5% glycerol, 0.5% TritonX-100, and EDTA-free cocktail complete protease inhibitor (Roche)]. Lysate supernatant was incubated with EZ red α-HA beads (Sigma-Aldrich) overnight, and beads were washed with lysis buffer and boiled in 2× Laemmli buffer. PAGE, transfer, and Western blot were performed according to standard protocols. In the RabGGTβ competition assay, we performed coIP using α-V5 beads (Sigma-Aldrich) instead. Primary antibodies were used at the following dilution: rabbit α-HA 1∶2,000 (Abcam), rabbit α-FLAG antibody 1∶2,000 (Abcam), mouse α-FLAG (M2, Sigma-Aldrich) 1∶1,000, mouse α-V5 1∶5,000 (Invitrogen), mouse α-Actin 1∶1,000 (MP Biomedicals), and mouse α-Rab11 1∶500 (BD Biosciences). Goat HRP-conjugated secondary antibodies were used at 1∶2,000 dilution (Jackson ImmunoResearch). Membranes were developed using Western Lightning Plus-ECL (PerkinElmer) followed by X-ray film (Thermo Scientific) detection.

### Rab Protein Purification and *In Vitro* Rab Prenylation Assay

GST, GST–Rab1, and GST–Rab11 were purified from BL21 pLys (Invitrogen): 25 ml overnight cultures were diluted to 250 ml with LB medium and kept growing at 37°C until OD_600_ reached 0.5∼0.7. GST, GST–Rab1, and GST–Rab11 proteins were induced at 37°C for 3 h by adding IPTG at a final concentration of 0.1 mM. Bacteria were then lysed using CelLytic express (Sigma-Aldrich) according to the manufacturer's instructions. Supernatant was incubated with Glutathione Sepharose 4B (GE healthcare) at 4°C for 2 h and then the beads were washed with 50 mM HEPES pH 8.0 buffer by inversion for three times. GST fusion proteins were eluted with 10 mM glutathione in 50 mM HEPES pH 8.0 buffer. Protein concentration was measured using Bradford method (BioRad). We used 0.3 µM GST–Rab1 and 3 µM GST–Rab11 as substrates in the prenylation assays with 0.3 µM and 3 µM GST as negative controls, respectively. On the other hand, all components of the RabGGT complex (His–RabGGTβ–V5, FLAG–REP, and HA, HA–Temp, or HA–RabGGTα) were transfected to S2 cells as described in the previous section. Cells were lysed on ice in prenylation buffer [50 mM HEPES, pH 7.2, 50 mM MaCl, 2 mM MgCl_2_, 0.01% Triton X-100, and EDTA-free cocktail complete protease inhibitor (Roche)] [60] using syringes. Supernatant was then incubated with EZ red α-HA beads (Sigma-Aldrich) for 4 h at 4°C, and beads were washed with prenylation buffer. We added HA, HA–Temp, or HA–RabGGTα binding beads together with GST control or GST–Rab substrates, 5 µM biotin-labeled lipid precursor (B-GPP; Jena Bioscience), 2 mM DTE, and 20 mM GDP. The prenylation assays were carried out on beads at 25°C for 1 h. Reactions were stopped by adding 2× Laemmli buffer. Western blot was performed as described in the previous section with 5% BSA as blocking solution and Streptavidin–HRP 1∶50,000 (Jackson ImmunoResearch) for Biotin detection.

## Supporting Information

Figure S1 **Schematic diagram and features of the three well-characterized PPTs.** PPTs are heterodimers of α and β subunits. They can be subdivided into three types: FT, GGTI, and GGTII (also called RabGGT) based on the lipid length and target motif present on their substrate proteins. All these genes are conserved from yeast to human. Because the α subunits of PPTs contain PPTA subunit repeat motifs, the FTα is also called PTAR2 and RabGGTα is also called PTAR3. PPTs add prenyl lipids (15-carbon farnesyl or 20-carbon geranylgeranyl group) via a thioether linkage (-C-S-C-) to a cysteine residue of soluble proteins. FT and GGTI share the same α subunit for substrate recognition and have a unique β subunit. They recognize the CaaX motif (C for cysteine residue, a for aliphatic residues, X can be a wide range of residues) in the C-terminal region of various substrates, including lamins as well as many small GTPases such as Ras and Rho. On the other hand, GGTII does not require a specific C-terminal motif for recognition. Because all of its substrates identified to date are Rab proteins, it is also called RabGGT. It requires assistance from REP to recruit Rab substrates. The target motif on most of the Rab proteins contains two Cysteine residues, and both are conjugated with geranylgeranyl groups.
(TIF)

Figure S2 **The cell fate transformation defect is not related to apoptosis in** ***temp*** **mutant clones.** (A–B) At 27 h APF, there are some apoptotic cells (Caspase3-positive cells) in some *temp^A^* mutant clones (A). However, there is no obvious apoptosis in *temp^B^* mutant clones (B). (C–D) When an anti-apoptotic protein, p35, is overexpressed in *temp^A^* mutant clones (24 h APF) to suppress the apoptotic effect (C), we observe no obvious differences in phenotype (D) when we compare these clones with *temp* mutant clones without p35 expression. Scale bars, 5 µm.
(TIF)

Figure S3 **HA–Temp protein is localized diffusedly throughout the cytoplasm.** HA–*temp* cDNA is expressed using dpp–Gal4 in the wing disc and HA–Temp is dispersed in the cytoplasm and does not seem to localize to any particular subcellular area. Scale bars, 5 µm.
(TIF)

Figure S4 **Accumulation of Sca in** ***temp*** **mutant is not restricted to notum ESOs.** (A) Sca accumulates in the *temp* mutant ESOs at the anterior of wing margin in third instar wing imaginal discs. (B) Single section: Sca accumulates in *temp* mutant R8 photoreceptor cells in the third instar larval eye discs. (B′) Projection of (B). (C) In the *temp* mutant clones, the expression level of *sca*–*lacZ* does not change, whereas the protein level of Sca is up-regulated in sensory organs at the anterior of wing margin during third instar larval stage. (D) In the *temp* mutant clones, the expression level of *sca*–*lacZ* does not change and the protein level of Sca is up-regulated in R8 cells in the third instar larval eye discs. (E) In *temp* mutant ESO, Sca puncta largely colocalize with GRASP, which locates at both ER exit site (tER) and cis-Golgi compartments. Scale bars, 5 µm.
(TIF)

Figure S5 **The localization of Dl, but not Notch or** ***D*** **E-cadherin, is altered in** ***temp*** **mutant ESOs.** (A) Total level of Notch (stained for NICD) is not changed in *temp* mutant clones. (B) Total level of Notch (stained for Notch extracellular domain) is not changed in *temp* mutant clones. (C) Extracellular level of Notch (stained for Notch extracellular domain without permeabilization) is not changed in *temp* mutant clones. (D) Many *temp* mutant ESOs exhibit increased total level of Dl puncta (yellow arrows and white arrows) compared to wt ESOs (red arrows). Dl in some *temp* mutant ESOs still localizes to the plasma membrane (white arrows), whereas Dl in other *temp* mutant ESOs can only be found intracellularly (yellow arrows). (E) Localization of *D*E-cadherin, an apically enriched transmembrane protein, is not affected in *temp* mutant clones. Asterisks (*) indicate folds in the notum where lower levels are artifactual. Scale bars, 5 µm.
(TIF)

Figure S6 **Dysfunction of Rab1 and Rab11 phenocopies loss-of-function of** ***temp*** **.** (A) Sca accumulates in *rab1* mutant sensory organs in the third instar larval wing disc, similar to the *temp* mutant phenotype. The *rab1* clones are mostly cell lethal even when the neighboring tissue has the competitive disadvantage of having a *Minute* mutation. (B) Single section: Overexpression of *Rab1DN* causes accumulation of both Dl and Sca on the notum. White arrow, *RablDN* expressing ESO; yellow arrow, control ESO. Note that the *RablDN*-expressing ESO in the upper region exhibits a severe Sca accumulation, which can also occasionally be observed in *temp* mutant ESOs, whereas *RablDN*-expressing ESO in the lower region exhibits a less severe Sca accumulation (puncta) similar to what is usually observed in *temp* mutant ESOs. (C) Adult notum: Overexpression of *Rab1DN* on the notum causing minor balding occasionally (arrow). (D) Western blot: Endogenous expression level of Rab11 is not altered in *temp^A^* and *temp^D^* mutant larvae compared to mutant larvae with a genomic rescue transgene (control). (E) Single section: Overexpression of *Rab11DN* does not affect the expression of Sca in the notum. White arrow, *Rab11DN*-expressing ESO; yellow arrow, wt ESO. (F) Single section: In *Rab11DN*-expressing cells, Dl is mislocalized in the middle plane of the cells. Scale bars, 5 µm.
(TIF)

Figure S7 **Overexpression of constitutively active form of Rab1 and Rab11 does not restore the** ***temp*** **mutant phenotypes.** (A–B) Overexpression of constitutively active forms of Rab1 (Rab1CA) either in control (*iso19A*) or *temp* mutant clones at 16–18 h APF: (A) Rab1CA overexpression in the control clones does not cause any phenotypes. (B) Rab1CA overexpression in the *temp* mutant clones does not rescue Sca accumulation. (C–D) Overexpression of constitutively active forms of Rab11 (Rab11CA) in either control (*iso19A*) or *temp* mutant clones at 27 h APF: (C) Rab11CA overexpression in control clones does not cause phenotypes. (D) Rab11CA overexpression in the *temp* mutant clones does not rescue the cell fate changes. Scale bars, 5 µm.
(TIF)

Figure S8 ***temp*** **mRNA expression is highly enriched in the nervous system.** The mRNA expression patterns of *temp* (*CG3073*) and *RabGGTα* (*CG12007*) are quite different in both larval and adult stages based on FlyAtlas data [65]. *RabGGTα* mRNA is transcribed at moderate level ubiquitously and *temp* mRNA is expressed highly in the nervous system, suggesting that Temp plays a role in the nervous system. This figure is adapted and modified from Flybase (http://flybase.org/) [66].
(TIF)

Table S1 **Sca accumulates in GM130-positive compartments in both** ***temp*** **mutant and** ***Rab1DN*** **-expressing notum clones.** We used MARCM to express ER–YFP, Golgi–CFP, FYVE–GFP, and LAMP-1–GFP in *temp* mutant or wt clones. For other markers, we performed coimmunostaining using specific antibodies (see Materials and Methods). ER, endoplasmic reticulum. +, colocalization; +/−, minor colocalization; −, no obvious colocalization; N/A, not tested.
(TIF)

Table S2 **Summary of Rab screen for Sca accumulation and balding.** ER, endoplasmic reticulum; EE, early endosome; PM, plasma membrane; LE, late endosome; TGN, *trans* Golgi network; RE, recycling endosome. +, positive, +/−, minor phenotype; −, no obvious phenotype; ND, not determined.
(TIF)

## Abbreviations

a.a.: amino acid
APF: after puparium formation
ARS: apical actin-rich
CA: constitutively active
CADASIL: cerebral autosomal dominant arteriopathy with subcortical infarcts and leukoencephalopathy
co-IP: co-immunoprecipitation
Dl: Delta
DN: dominant negative
ELAV: Embryonic Lethal Abnormal Vision
EMS: ethyl methanesulfonate
ESO: external sensory organ
FBS: fetal bovine serum
FT: farnesyltransferase
GGT: geranylgeranyltransferase
HA: Hemagglutinin
hPTAR1: human PTAR1
iso19A: isogenized y w FRT19A
MARCM: mosaic analysis with a repressible cell marker
NICD: Notch intracellular domain
PPT: protein prenyltransferase
PTAR1: PPTA containing protein 1
PTAR2: PPTA containing protein 2
PTAR3: PPTA containing protein 3
RabGGT: Rab geranylgeranyltransferase
REP: Rab escort protein
sca: scabrous
SCD: spondylocostal dysostosis
sens: senseless
SOP: sensory organ precursor
Su(H): Suppressor of Hairless
Syx16: Syntaxin 16
temp: tempura
Ttk: Tramtrack
wt: wild-type
