# Supplementary figures and images for: Drosophila Tempura, a Novel Protein Prenyltransferase α Subunit, Regulates Notch Signaling Via Rab1 and Rab11
Source: PLoS Biol. 2014 Jan 28;12(1):e1001777. doi: 10.1371/journal.pbio.1001777 (PMC3904817; doi:10.1371/journal.pbio.1001777)

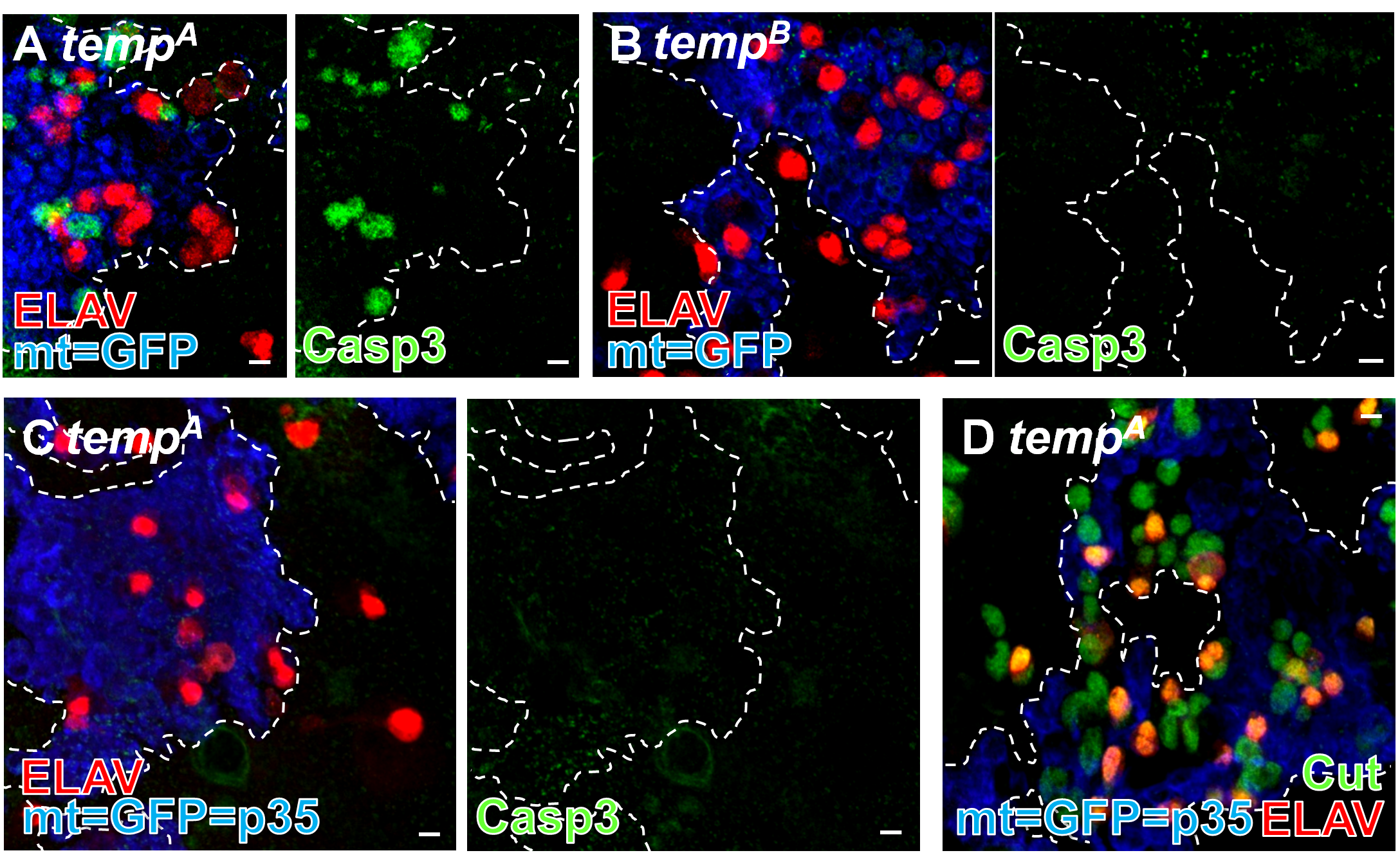

Supplement: Figure S2 — The cell fate transformation defect is not related to apoptosis in temp mutant clones. (A–B) At 27 h APF, there are some apoptotic cells (Caspase3-positive cells) in some tempA mutant clones (A). However, there is no obvious apoptosis in tempB mutant clones (B). (C–D) When an anti-apoptotic protein, p35, is overexpressed in tempA mutant clones (24 h APF) to suppress the apoptotic effect (C), we observe no obvious differences in phenotype (D) when we compare these clones with temp mutant clones without p35 expression. Scale bars, 5 µm. (TIF) [file pbio.1001777.s002.tif]

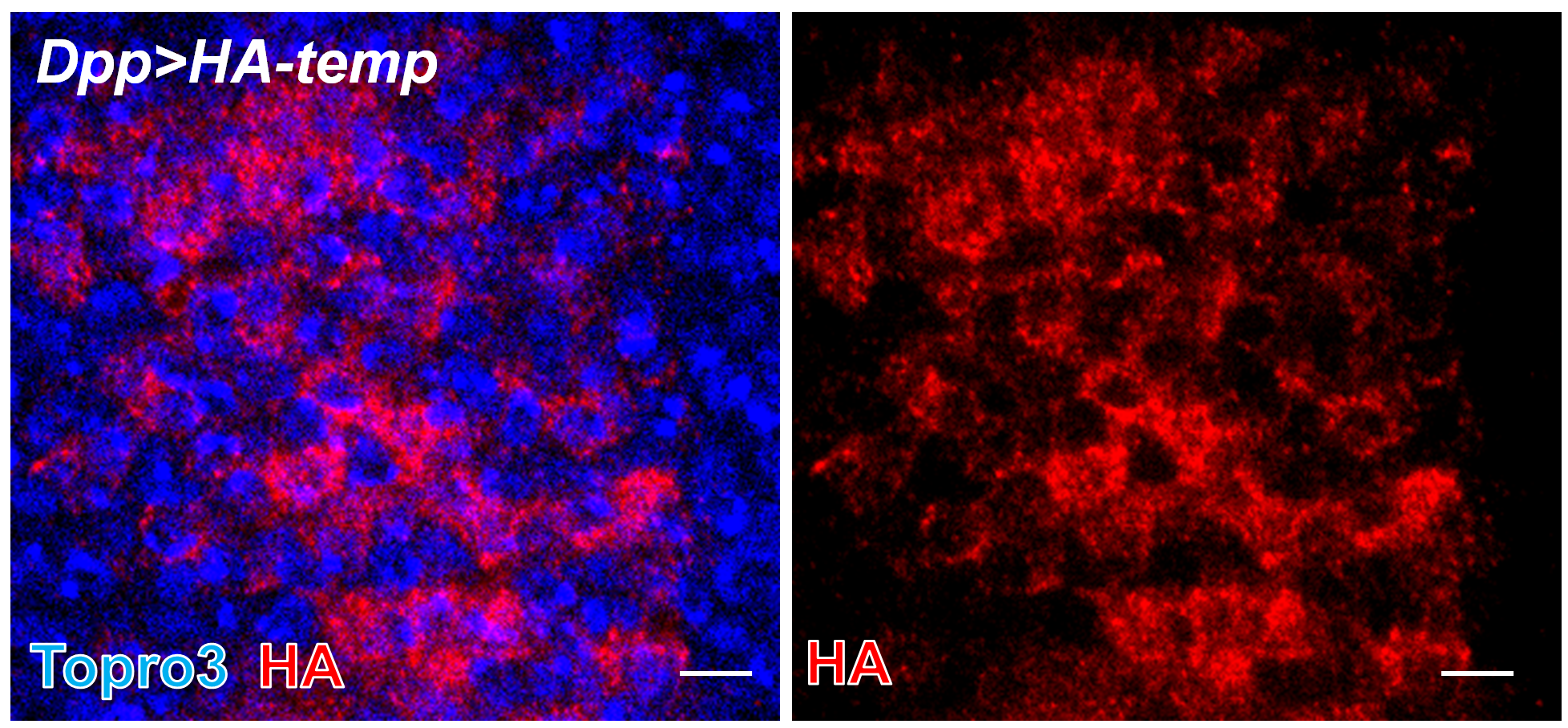

Supplement: Figure S3 — HA–Temp protein is localized diffusedly throughout the cytoplasm. HA–temp cDNA is expressed using dpp–Gal4 in the wing disc and HA–Temp is dispersed in the cytoplasm and does not seem to localize to any particular subcellular area. Scale bars, 5 µm. (TIF) [file pbio.1001777.s003.tif]

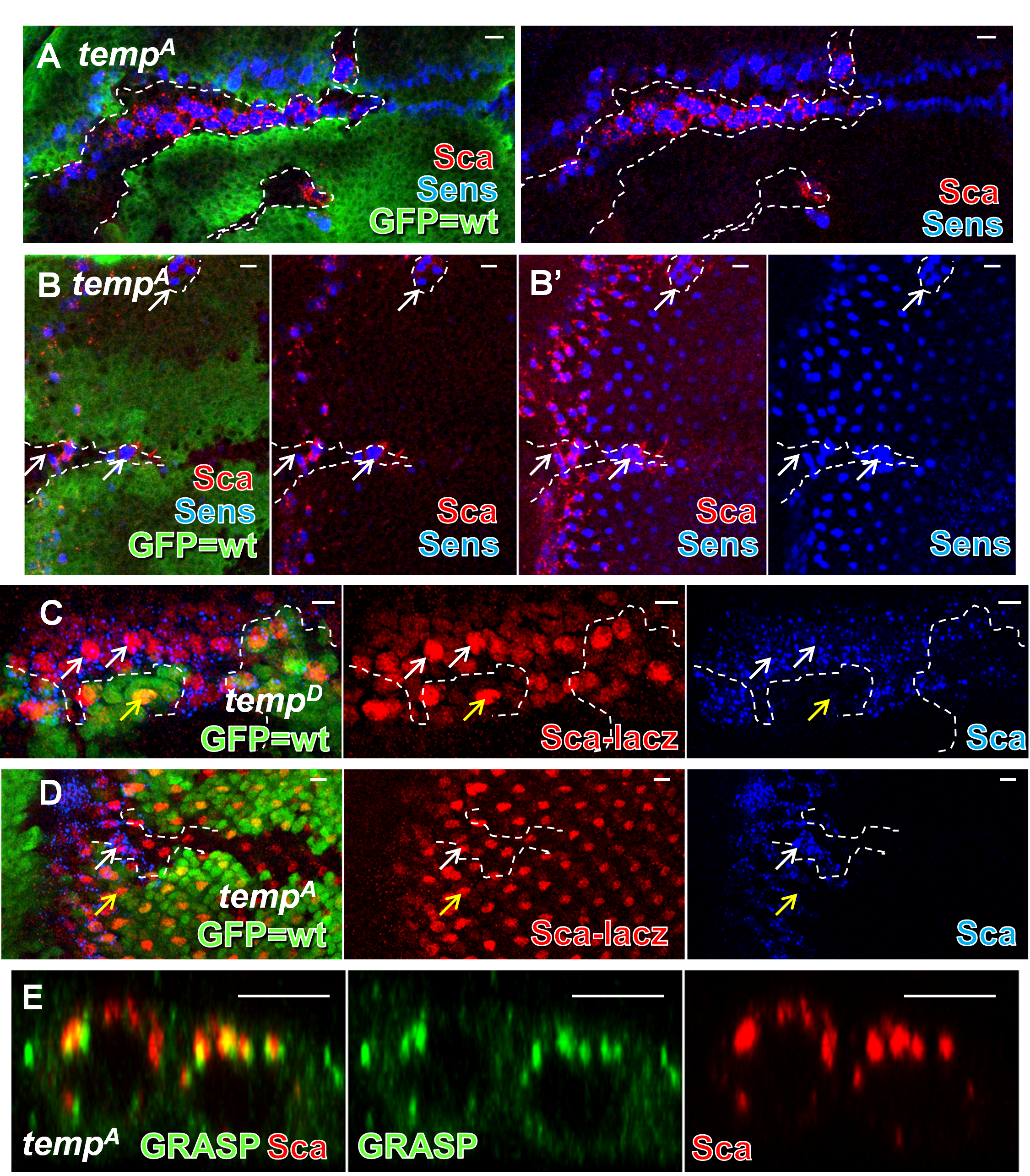

Supplement: Figure S4 — Accumulation of Sca in temp mutant is not restricted to notum ESOs. (A) Sca accumulates in the temp mutant ESOs at the anterior of wing margin in third instar wing imaginal discs. (B) Single section: Sca accumulates in temp mutant R8 photoreceptor cells in the third instar larval eye discs. (B′) Projection of (B). (C) In the temp mutant clones, the expression level of sca–lacZ does not change, whereas the protein level of Sca is up-regulated in sensory organs at the anterior of wing margin during third instar larval stage. (D) In the temp mutant clones, the expression level of sca–lacZ does not change and the protein level of Sca is up-regulated in R8 cells in the third instar larval eye discs. (E) In temp mutant ESO, Sca puncta largely colocalize with GRASP, which locates at both ER exit site (tER) and cis-Golgi compartments. Scale bars, 5 µm. (TIF) [file pbio.1001777.s004.tif]

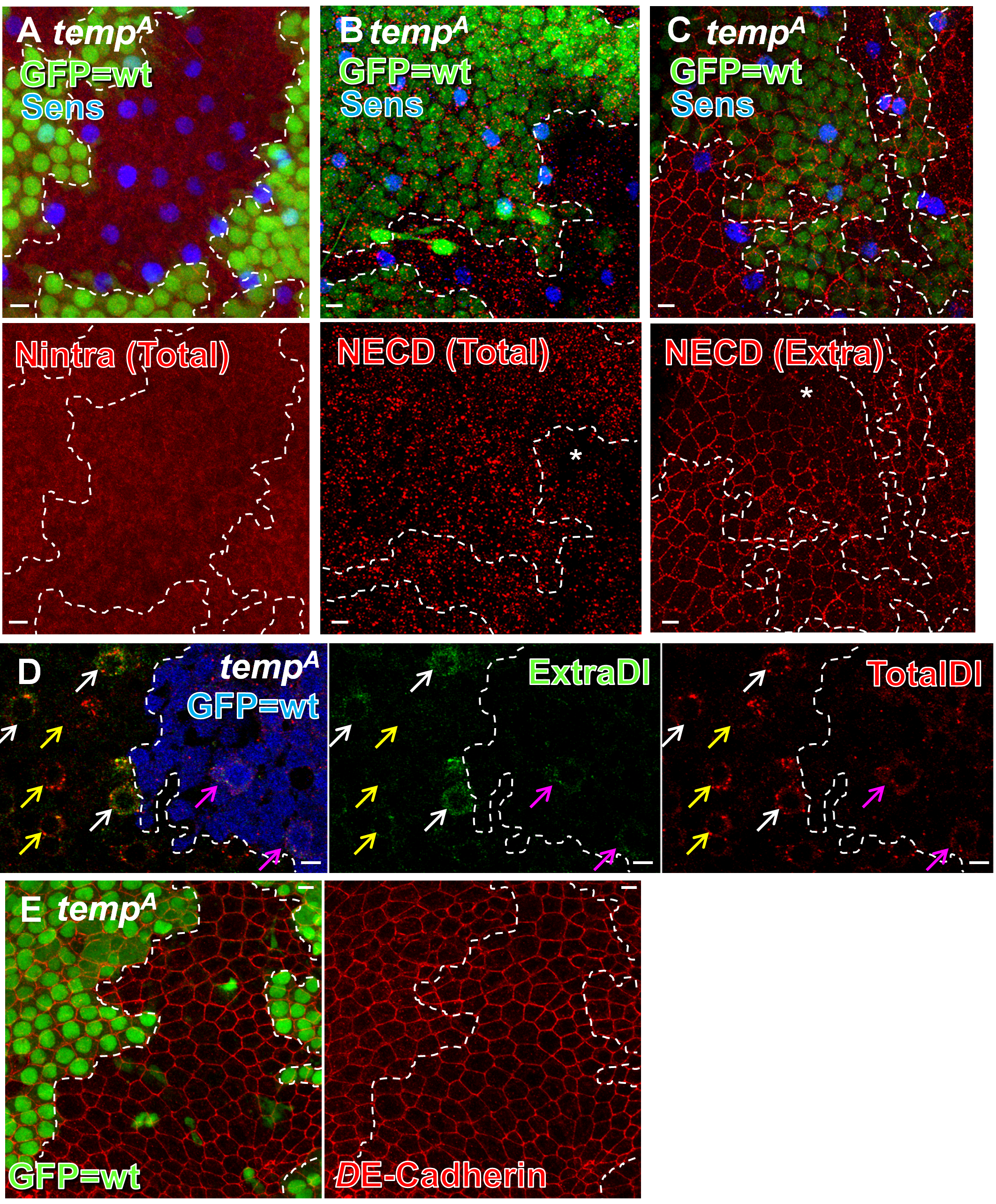

Supplement: Figure S5 — The localization of Dl, but not Notch or D E-cadherin, is altered in temp mutant ESOs. (A) Total level of Notch (stained for NICD) is not changed in temp mutant clones. (B) Total level of Notch (stained for Notch extracellular domain) is not changed in temp mutant clones. (C) Extracellular level of Notch (stained for Notch extracellular domain without permeabilization) is not changed in temp mutant clones. (D) Many temp mutant ESOs exhibit increased total level of Dl puncta (yellow arrows and white arrows) compared to wt ESOs (red arrows). Dl in some temp mutant ESOs still localizes to the plasma membrane (white arrows), whereas Dl in other temp mutant ESOs can only be found intracellularly (yellow arrows). (E) Localization of DE-cadherin, an apically enriched transmembrane protein, is not affected in temp mutant clones. Asterisks (*) indicate folds in the notum where lower levels are artifactual. Scale bars, 5 µm. (TIF) [file pbio.1001777.s005.tif]

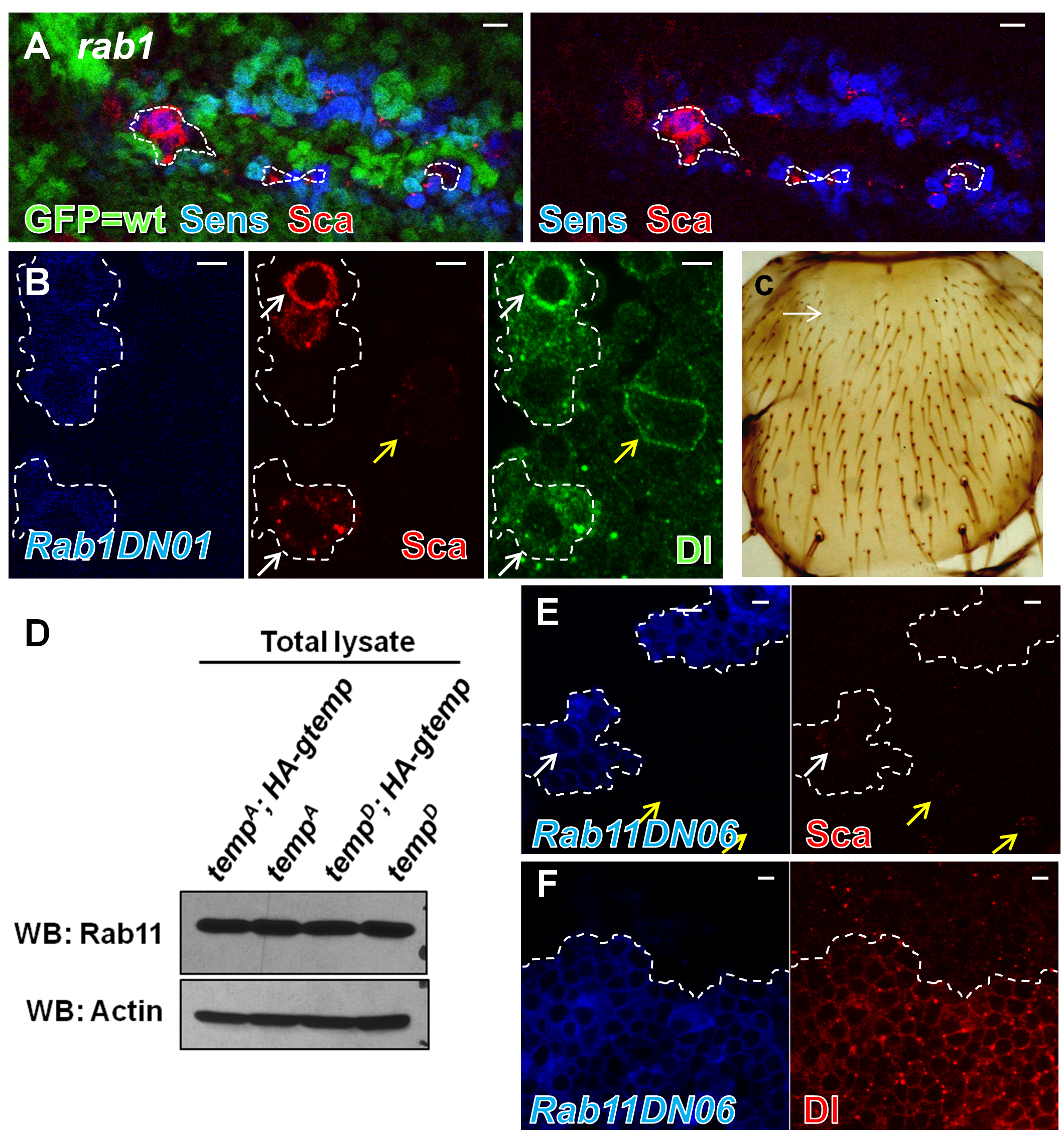

Supplement: Figure S6 — Dysfunction of Rab1 and Rab11 phenocopies loss-of-function of temp . (A) Sca accumulates in rab1 mutant sensory organs in the third instar larval wing disc, similar to the temp mutant phenotype. The rab1 clones are mostly cell lethal even when the neighboring tissue has the competitive disadvantage of having a Minute mutation. (B) Single section: Overexpression of Rab1DN causes accumulation of both Dl and Sca on the notum. White arrow, RablDN expressing ESO; yellow arrow, control ESO. Note that the RablDN-expressing ESO in the upper region exhibits a severe Sca accumulation, which can also occasionally be observed in temp mutant ESOs, whereas RablDN-expressing ESO in the lower region exhibits a less severe Sca accumulation (puncta) similar to what is usually observed in temp mutant ESOs. (C) Adult notum: Overexpression of Rab1DN on the notum causing minor balding occasionally (arrow). (D) Western blot: Endogenous expression level of Rab11 is not altered in tempA and tempD mutant larvae compared to mutant larvae with a genomic rescue transgene (control). (E) Single section: Overexpression of Rab11DN does not affect the expression of Sca in the notum. White arrow, Rab11DN-expressing ESO; yellow arrow, wt ESO. (F) Single section: In Rab11DN-expressing cells, Dl is mislocalized in the middle plane of the cells. Scale bars, 5 µm. (TIF) [file pbio.1001777.s006.tif]

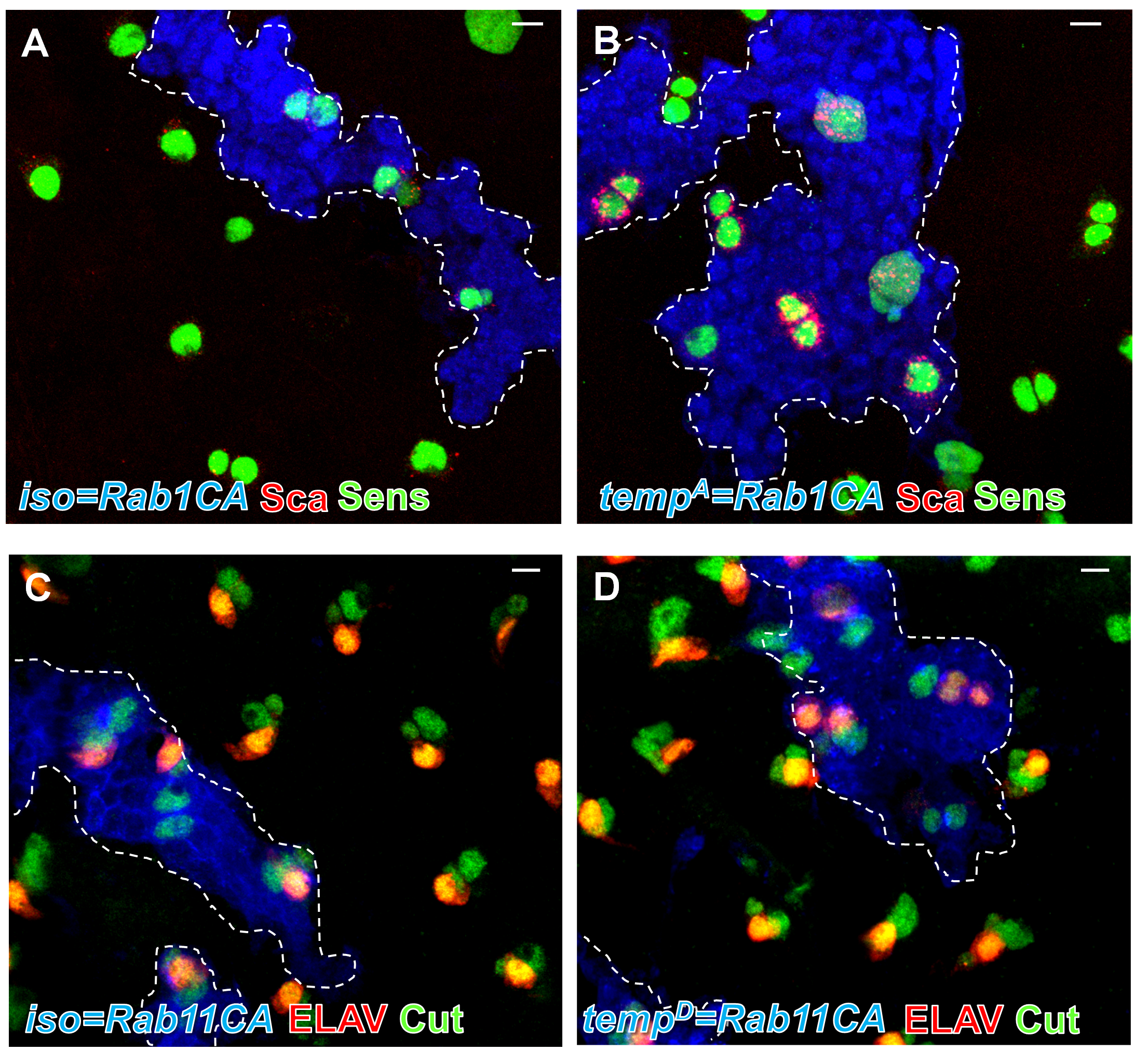

Supplement: Figure S7 — Overexpression of constitutively active form of Rab1 and Rab11 does not restore the temp mutant phenotypes. (A–B) Overexpression of constitutively active forms of Rab1 (Rab1CA) either in control (iso19A) or temp mutant clones at 16–18 h APF: (A) Rab1CA overexpression in the control clones does not cause any phenotypes. (B) Rab1CA overexpression in the temp mutant clones does not rescue Sca accumulation. (C–D) Overexpression of constitutively active forms of Rab11 (Rab11CA) in either control (iso19A) or temp mutant clones at 27 h APF: (C) Rab11CA overexpression in control clones does not cause phenotypes. (D) Rab11CA overexpression in the temp mutant clones does not rescue the cell fate changes. Scale bars, 5 µm. (TIF) [file pbio.1001777.s007.tif]

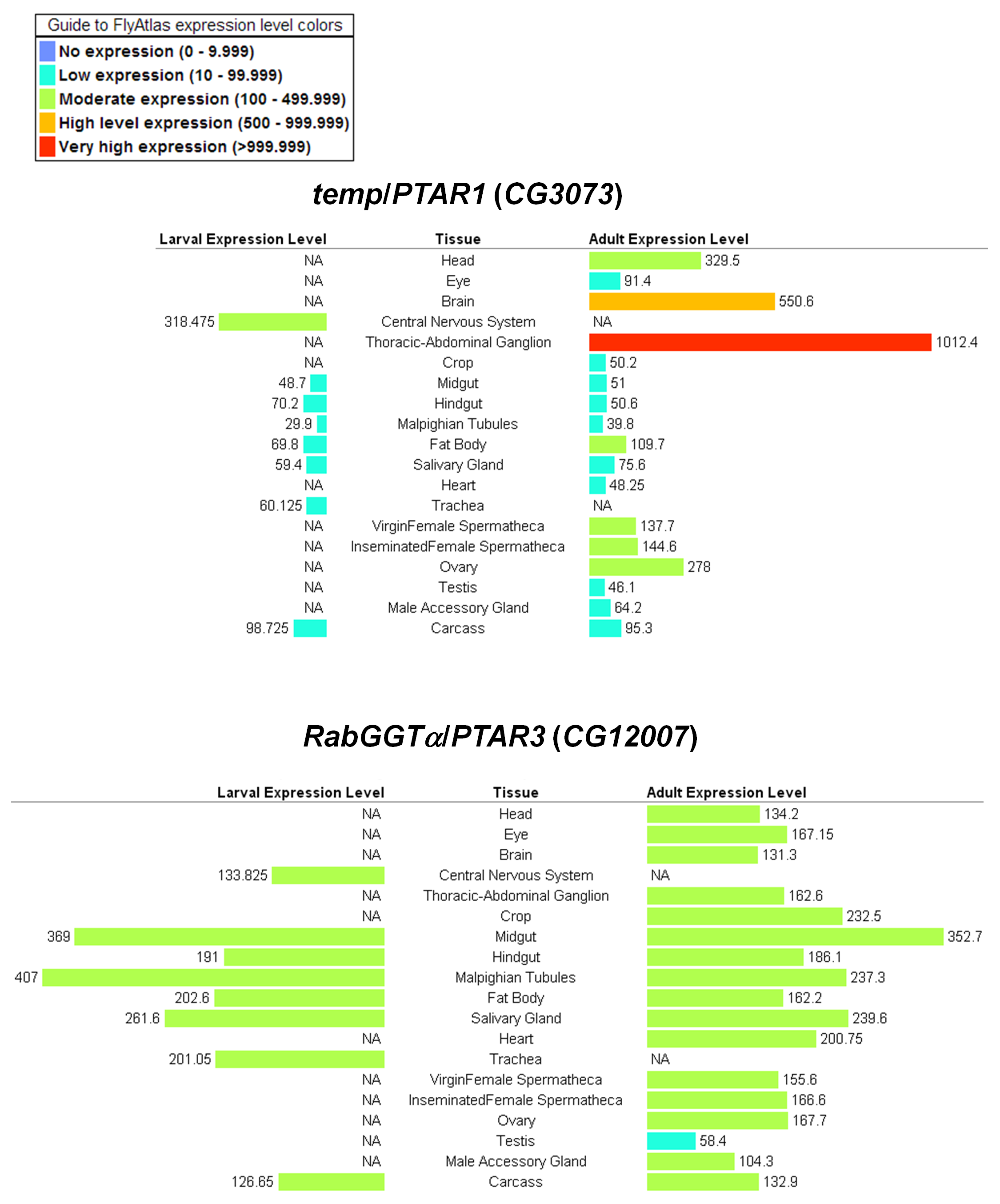

Supplement: Figure S8 — temp mRNA expression is highly enriched in the nervous system. The mRNA expression patterns of temp (CG3073) and RabGGTα (CG12007) are quite different in both larval and adult stages based on FlyAtlas data [65]. RabGGTα mRNA is transcribed at moderate level ubiquitously and temp mRNA is expressed highly in the nervous system, suggesting that Temp plays a role in the nervous system. This figure is adapted and modified from Flybase (http://flybase.org/) [66]. (TIF) [file pbio.1001777.s008.tif]

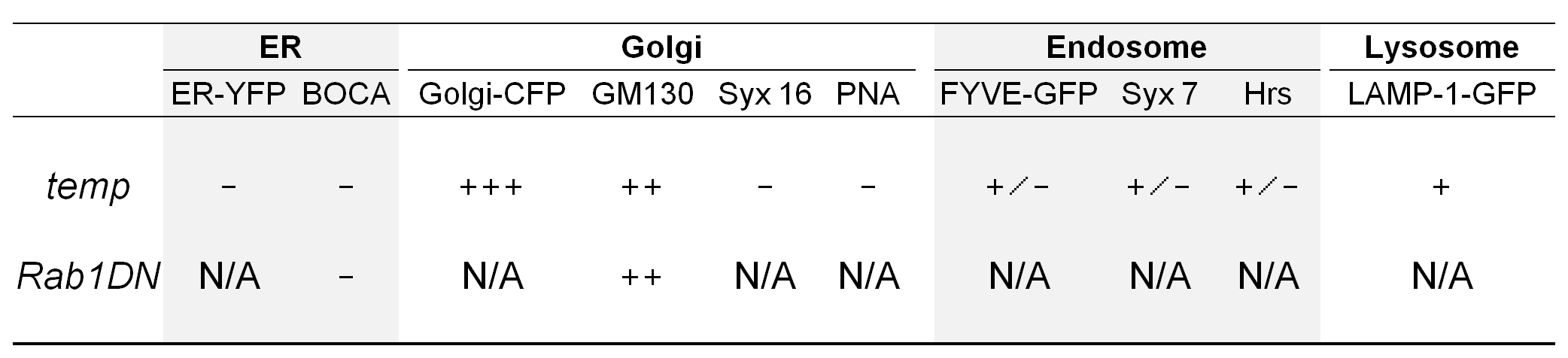

Supplement: Table S1 — Sca accumulates in GM130-positive compartments in both temp mutant and Rab1DN -expressing notum clones. We used MARCM to express ER–YFP, Golgi–CFP, FYVE–GFP, and LAMP-1–GFP in temp mutant or wt clones. For other markers, we performed coimmunostaining using specific antibodies (see Materials and Methods). ER, endoplasmic reticulum. +, colocalization; +/−, minor colocalization; −, no obvious colocalization; N/A, not tested. (TIF) [file pbio.1001777.s009.tif]

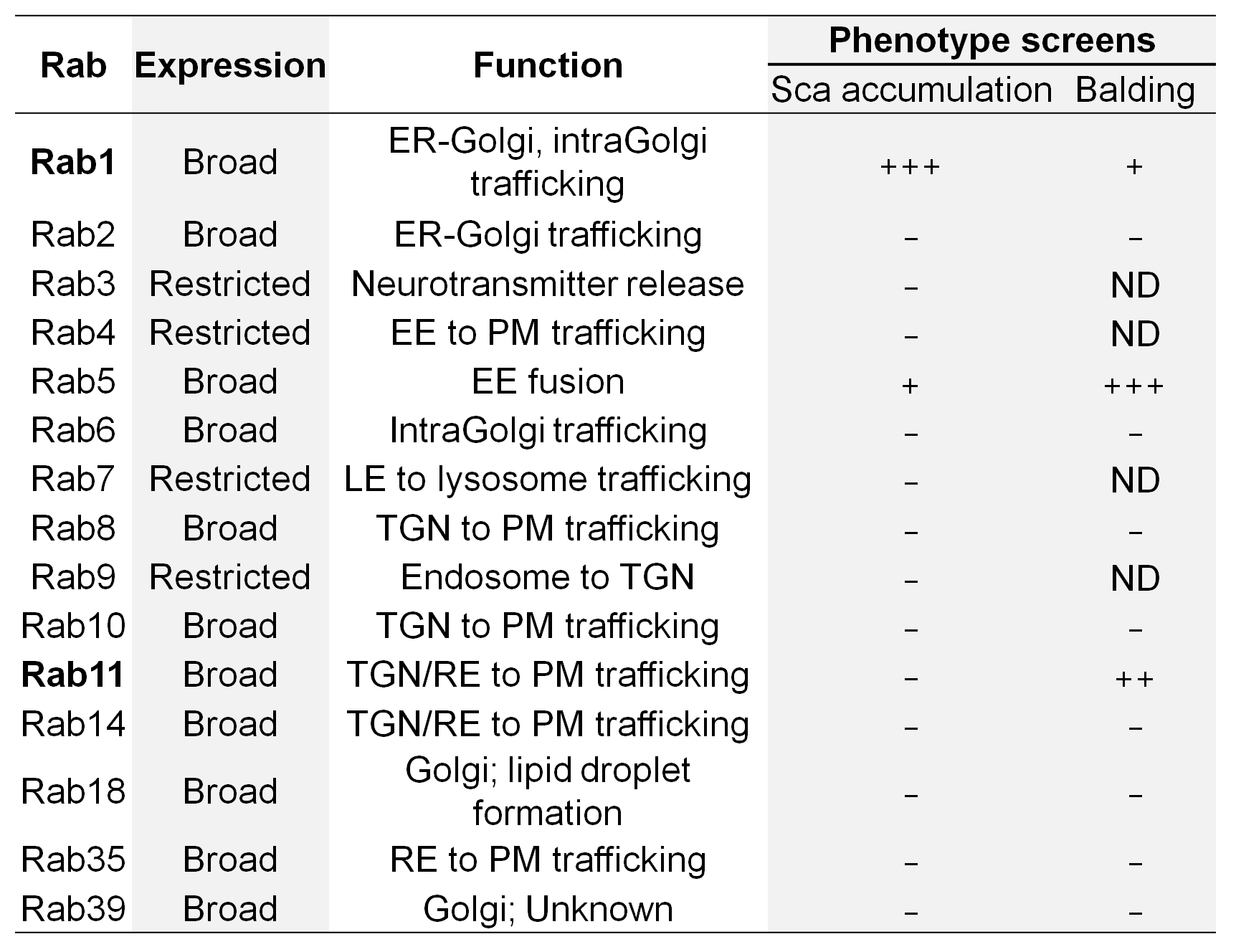

Supplement: Table S2 — Summary of Rab screen for Sca accumulation and balding. ER, endoplasmic reticulum; EE, early endosome; PM, plasma membrane; LE, late endosome; TGN, trans Golgi network; RE, recycling endosome. +, positive, +/−, minor phenotype; −, no obvious phenotype; ND, not determined. (TIF) [file pbio.1001777.s010.tif]
